# Isotherm, kinetics and ANN analysis of methylene blue adsorption onto nitrogen doped *Ulva lactuca* Biochar

**DOI:** 10.1038/s41598-025-92973-y

**Published:** 2025-03-27

**Authors:** Amany G. M. Shoaib, Murat Yılmaz, Amany El Sikaily, Mohamed A. Hassaan, Mohamed A. El-Nemr, Ahmed El Nemr

**Affiliations:** 1 National Institute of Oceanography and Fisheries (NIOF), Kayet Bey, Elanfoushy, Alexandria Egypt; 2 BahçE Vocational School, Department of Chemistry and Chemical Processing Technologies, Osmaniye Korkut Ata University, Osmaniye, 80000 Türkiye Turkey; 3 Department of Chemical Engineering, Faculty of Engineering, Minia University, Minia, 61519 Egypt; 4 The Higher Canal Institute of Engineering and Technology, Al Salam 1 - Abu Bakr Al Siddiq Street, Suez, Egypt

**Keywords:** Green algae, *Ulva lactuca*, Methylene blue, Ammonia-modified Biochar, Adsorption, Nitrogen-doped Biochar, Chemical engineering, Environmental chemistry

## Abstract

This study investigates the removal of methylene blue (MB) dye from aqueous solutions using a novel adsorbent, green algae (*Ulva lactuca*)-derived biochar-ammonia (NDULB), produced through activation with 85% sulfuric acid and hydrothermal treatment with ammonium hydroxide. The characterization of NDULB was carried out through various techniques, including BET surface area analysis and scanning electron microscopy, confirming its high surface area and effective porosity for dye adsorption. This work thoroughly examines the effects of initial MB dye concentration, solution pH, contact time, and NDULB dose on adsorption. The adsorption data were modeled using Langmuir, Freundlich, Tempkin, and Dubinin-Radushkevich isotherms, with the Freundlich model showing the best fit, indicating multilayer adsorption on a heterogeneous surface. According to the investigation’s findings, with an initial MB concentration of 200 ppm and an NDULB dosage of 1.25 g L^−1^, the adsorption capacity at equilibrium (*q*_*e*_) is 966.31 mg g^−1^. Kinetic analysis revealed that the pseudo-second-order model provided the best fit for the experimental data, suggesting chemisorption as the dominant adsorption mechanism. The artificial neural network modeling has been studied and reported. The study clarifies the effects of multiple variables on adsorption, which might lead to key insights to enlighten the development of effective wastewater treatment strategies. The study demonstrates that NDULB offers a promising, sustainable alternative for MB dye removal in wastewater treatment, with significant implications for large-scale application.

## Introduction

Many sectors use dyes to colour their products, including paper, food, rubber, plastics, carpets, cosmetics, and textiles^1–3^. When various colouring materials are produced and consumed, up to 10% of the utilized colours are released as wastewater, which leads to health^4,5^and environmental^6^issues. Because dyeing wastewater discharge contains poisonous heavy metals, high acidic pH levels, chemical oxygen demand (COD), and other harmful pollutants, it is referred to as highly polluted wastewater^7–9^.

The azo dye group is the most significant and abundant dye produced in the industry. It is identified by the presence of azo-groups (-N = N-) about aromatic rings (benzene and/or naphthalene)^10^. Because of its extended water life, methylene blue (MB) dye is one of the azo dyes known to be among the most dangerous^11–13^. Owing to its considerable toxicity, MB dye adversely affects human health at certain concentrations. Its toxic, carcinogenic, and non-biodegradable properties present a significant threat to both human health and the ecosystem^14^. All countries have recently tightened their regulations to prevent this kind of wastewater with dyes from entering public waterways unless adequately treated. Numerous biological, physical, and chemical methods have been documented to remove dye from wastewater^5,15,16^. Numerous strategies were investigated to remove various pollutants from wastewater, including advanced oxidation for textile dye effluent treatment^17,18^, the synthesis of various composites for toxic metal-containing water treatment^19,20^, and the use of nanomaterials as a catalyst for the photodegradation of textile effluents^21,22^.

Adsorption is one of the best ways to remove dyes from wastewater among treatment procedures for dye wastewater since it doesn’t require a huge application area and can be done quickly and affordably^6^. Adsorption is also a technology that is still frequently adopted due to its user-friendly structure, remarkable efficiency, environmental sustainability, resistance to harmful chemicals, and adaptability^23–25^. However, the kind of functional groups on the adsorbent’s surface and its structural makeup significantly impact the adsorption process^26^. The different functional groups present on the adsorbent material’s surface significantly affect the maximum adsorption capacity and the adsorption mechanism of various contaminants^27^. Materials with a high adsorption capacity range from novel solutions like biochar or modified clays to conventional activated carbon^28–30^. It has been noted that biochar can remove both organic and inorganic contaminants from industrial effluents, highlighting its adaptability and efficiency in wastewater treatment^30–32^.

Moreover, successful separation from the aqueous phase has been investigated using engineered biochar incorporating a suitable magnetic medium, increasing its potential for helpful use in wastewater treatment^9,11,33,34^. Many studies have investigated the use of biochar in water and wastewater treatment, demonstrating the material’s strong ability to sorb a wide range of pollutants, including hydroquinone^35^. Researchers have also investigated the use of biochar in biofiltration systems to eliminate pharmaceuticals, personal hygiene products, and nutrients from effluent together with microorganisms. This demonstrates the range of toxins that biochar may eliminate^36^. The effect of N_2_modification on adsorption capacity was evidenced by nitrogen-doped hierarchical porous biochar derived from maize stalks, which exhibited enhanced phenol removal^37^. Furthermore, modification techniques, including HNO_3_, NaOH, and Na_2_S, have been shown to strengthen biochar’s ability to adsorb Mn(II) particles^38^. Furthermore, the utilization of Ca-rich biochars made from leftover mushroom substrates has demonstrated the superior adsorption qualities of cationic dyes, suggesting the potential for specific alterations for targeted adsorption applications^39^. The use of H_2_SO_4_ (sulfuric acid) and NH_4_OH (ammonium hydroxide) as activating agents is justified due to their ability to modify the surface properties of biochar and enhance its adsorption capacity. H_2_SO_4_ acts as a strong acid, promoting the formation of a highly porous structure and introducing functional groups on the surface, which increases the biochar’s surface area and reactivity. NH_4_OH, on the other hand, helps to neutralize the surface and introduce nitrogen-rich groups, further improving the adsorption of organic pollutants like methylene blue. Together, these agents create a biochar with optimized properties for efficient dye removal from aqueous solutions^1,40,41^.

Within the environmental remediation field, using biochar made from the green algae *Ulva lactuca* (*U. lactuca*) to adsorb contaminants from aqueous solutions is a novel and environmentally beneficial strategy. Since green algae may be grown in various water bodies- even ones that are unsuitable for other uses-it serves as a sustainable and renewable source material for biochar^42–45^. The large surface area and porosity of algae-based biochar, particularly when modified, may have considerably aided in the adsorption of pollutants, including organic compounds, colours, and heavy metals from wastewater^33,46–48^. Furthermore, it highlights how the algae-based biochar’s adsorption capability and surface chemistry were enhanced by the presence of N-functional groups^49^. These results highlight the green algae-derived modified biochar’s potential for effective pollutant adsorption.

An important and novel addition to the wastewater treatment field is the study of using modified biochar made from green algae, namely *U. lactuca*, to remove MB dye from wastewater. This study closes an important gap in the literature by focusing primarily on traditional, usually less sustainable methods of treating textile effluent. The investigation of a viable and efficient substitute employing biochar produced from *U. lactuca* constitutes the innovative aspect of this study. Green algae biochar offers an economical, eco-friendly, and renewable alternative to conventional adsorbents. This biochar has been modified to increase its adsorption capacity, especially for MB. This is a novel approach to target particular contaminants that are frequently present in textile effluent.

Hydrothermal conversion is a promising method for converting organic materials, such as algae or biomass, into valuable products like biochar, biofuels, and other chemicals. This process occurs in a water-rich environment under elevated temperatures and pressures, which accelerates the breakdown of complex organic compounds. The benefits of hydrothermal conversion include its ability to handle moist feedstocks without the need for pre-drying, its efficiency in producing high-value carbon-rich materials, and its potential to reduce waste and lower environmental impact. Furthermore, hydrothermal treatment can enhance the surface properties of biochars, making them more effective for applications such as wastewater treatment and carbon sequestration^25,50–53^.

Furthermore, using the readily available and underutilized biomass of green algae, *U. lactuca*, to produce biochar not only provides an effective solution for wastewater treatment but also aids in managing carbon sequestration and algal blooms. This study closes the gap by highlighting the ability of biochar changes to satisfy specific industrial effluent treatment requirements and offering a practical, scalable, and environmentally friendly strategy. Previous research has not thoroughly examined this aspect^1,43^.

Artificial neural networks are widely used as alternative mathematical techniques to address many problems with few adjustments. Because the adsorption process is intricate and nonlinear, researchers are urged to employ artificial neural networks (ANN) to anticipate the adsorption profile^54^. An appropriate experimental approach is crucial for assessing the impacts of important factors. The investigations are designed using the Artificial Neural Network (ANN). This method helps produce ideal parameters for experiment modeling and optimizing the removal of harmful wastewater^55,56^. Excellent prediction results are provided by the Artificial Neural Network model, which may be utilized to optimize^57–59^. In the adsorption of heavy metals using a natural biosorbent, artificial neural networks (ANNs) are utilized to simulate human perception in decision-making for complex, nonlinear, partial, and irrelevant information^60^. Environmental modeling has successfully employed the ANN technique to track nonlinear interactions between factors in intricate systems, such as medications and calcium alginate beads^61^.

In order to adsorb methylene blue from wastewater, this investigation will use biochar made from the green algae *U. lactuca* (GAUL) with concentrated H_2_SO_4_ and NH_4_OH. The novelty of this research lies in its exploration of green algae-based biochar-ammonia (NDULB) as an effective and sustainable adsorbent for the removal of methylene blue (MB) dye from wastewater. While biochars derived from various feedstocks have been widely studied for dye removal, this study is unique in its use of a green algae-based biochar activated with H_2_SO_4_ and NH_4_OH, which offers a sustainable and cost-effective alternative to traditional adsorbents. By investigating various factors such as adsorbent dosage, pH, contact time, and initial MB dye concentration, the research aims to optimize the adsorption process. The study’s goal is to provide valuable insights into the adsorption mechanisms and kinetics, with a focus on the Freundlich isotherm and PSOM kinetic models, to improve large-scale wastewater treatment systems. Ultimately, the findings support the development of sustainable and efficient water treatment technologies for industrial applications.

## Materials and methods

### Materials

Sodium Hydroxide (NaOH, Mwt = 39.997 g/mole), Sulfuric acid (H_2_SO_4_, 99%, Mwt = 98.079 g/mole), Hydrogen Chloride (HCl, 37%, Mwt = 36.46 g/mole), Sodium bicarbonate (NaHCO_3_, Mwt = 84 g/mole), Ammonia solution (NH_4_OH, 33%, Mwt = 35.04 g/mole). Methylene Blue (basic blue 9; C.I. 52,015, *λ*_max_ = 665 nm, M.F. = C_16_H_18_N_3_ClSxH_2_O, Mwt = 319.86 g/mole) was obtained from Honeywell Riedel-de Haën AG, Seelze-Hannover, Germany.

### Sampling

GAUL was collected from the Alexandria coastal area, thoroughly washed with tap and distilled water, and then dried first at room temperature overnight and subsequently in an oven at 120 °C. The dried GAUL was ground into a fine powder using an electric blender. The green powder was sieved through a 100-mesh screen before use. GAUL exhibited a density of 0.8179 g/mL, a moisture content of 15.3%, and an ash content of 35.12%.

### Preparation of stock solution

**Fig. 1:**
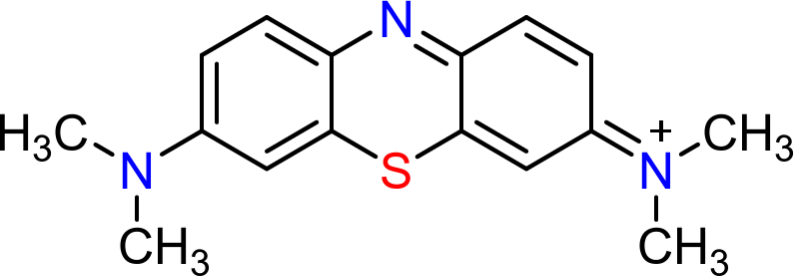
Chemical structure of MB dye (basic blue 9; C.I.52015, *λ*_max_ = 665 nm, MF = C_16_H_18_N_3_ClS.xH_2_O, Mwt = 319.86 g/mole).

A stock solution was prepared by dissolving 1.0 g of MB dye (Fig. 1) in 1000 mL of distilled water (DW). This stock solution was then diluted as needed for the standard calibration curve and absorption studies using a 1 cm optical path and a double-beam UV/visible spectrophotometer (PG instrument, model T80, United Kingdom) with glass cells.

### Preparation of NDULB

To create sulfur-doped *Ulva lactuca* biochar (SDULB), 300 g of dried GAUL was mixed with 600 mL 98% H₂SO₄ and 250 mL of H_2_O, followed by refluxing for 4 h. The mixture was then filtered, purified with DW, and rinsed with a 1% NaHCO_3_ solution to remove any remaining acid until the filtrate reached neutrality. Finally, it was rinsed with ethanol and dried for 24 h at 120 °C. 25 g of SDULB and 70 mL of ammonia solution were reacted for three hours at 140 °C in a hydrothermal system to create NDULB. The biochar was first labeled NDULB after being cooled, filtered, cleaned with DW and ethanol, and then dried for 24 h at 120 °C.

### Batch absorption method

Through a series of batch studies, the effectiveness of NDULB in removing MB dye was examined. Whereby batch equilibrium experiments were used to examine the impacts of starting MB dye concentration, contact time, and starting pH on the adsorption uptake. A set of Erlenmeyer flasks containing 100 mL of different starting MB dye starting concentrations (100, 125, 150, 175, and 200 mg/L) and different NDULB dosages (0.5, 0.75, 1.0, 1.25, and 1.5 g/L) were used for batch adsorption investigations at 25 °C. An isothermal shaker, set to 200 rpm and maintained at a constant temperature, was used until a specified endpoint. The concentration of the MB dye was measured utilizing a double-beam UV–visible spectrophotometer (PG equipment, model T80, UK). At *λ*_max_ = 665 nm, the wavelength reached its maximum. An initial MB dye concentration ranging from 100 to 200 mg/L was prepared to examine the impacts of contact time and starting dye concentration on adsorption uptake. The effect of pH on the removal process was examined by varying the initial pH between 2 and 12.

Additionally, the equilibrium data were fitted to the Temkin, Freundlich, and Langmuir isotherm models to investigate the adsorption isotherms. The kinetics of the removal process were examined in batch experiments utilizing the pseudo-first-order (PFOM), pseudo-second-order (PSOM), Elovich (EM), and intraparticle diffusion kinetic models (IPDM). The slope, intercept, *q*_cal_, *q*_exp_, and least-squares correlation coefficient (*R*^2^) were computed for every experiment to apply the specified models to the kinetic data.

The capacity of adsorption at equilibrium (*q*_e_) was determined by Eq. (1).

1 $$\:{q}_{e}=\frac{\left({C}_{0}-{C}_{e}\right)\:V}{m}\:$$

where the absorbent’s capability to absorb MB dye from water within a defined time is quantified as the absorption capacity (*q*_e_) (mg/g). The starting MB dye concentration is denoted as *C*_0_ (mg/L), while the residual MB dye concentration at equilibrium, after a specified period, is represented as *C*_e_ (mg/L). The MB dye elimination % from water may be calculated using Eq. (2).

2 $$\:Removal\:\%\:=\frac{\left({C}_{0}-{C}_{e}\right)\:}{{C}_{0}}\:\times\:100\:$$

### Characterization and instruments

A digital UV/visible spectrophotometer, a JENCO-6173 pH meter, and a JSOS-500 shaker were used. The attributes of NDULB were analyzed utilizing the required instrument. The X-ray diffraction (XRD) pattern of NDULB was obtained at an angle of 2*θ* utilizing an X-ray diffractometer (Ulitama IV, Rigaku, Tokyo, Japan). The shape and size of the biochar were investigated utilizing a field emission scanning electron microscope (SEM-JEOL, IT 200, Japan), operated at an acceleration voltage of 15.0 kV. The elemental contents of N, C, and O in the materials were detected by X-ray spectrometry (EDX) and CHNS/O elemental analysis. Thermo Scientific FlashSmart Elemental Analyser (EA) connected to Thermo Scientific MAS Plus Autosampler and EagerSmart Data Handling Software was used to perform the CHNS/O elemental analysis. The multipoint N_2_ adsorption Brunauer–Emmett–Teller (BET) technique determined specific surface area, pore size, and pore volume (BELSORP-Mini II, BEL Japan, Inc.). The functional groups on the sample surfaces were identified using a Fourier-transform infrared (FTIR) spectrometer (VERTEX 70) equipped with an ATR unit (V-100) in the range of 400–4000 cm⁻¹. The NDULB was exposed to breakdown at temperatures between 25 and 1000 °C, with a temperature rise rate of 5 °C every minute, utilizing a thermogravimetric analyzer (TGA) to perform approximations of the NDULB. The moisture %, ash, volatile matter, and fixed carbon were all calculated on a dry basis, summing to 100%. To evaluate the moisture content, NDULB samples were subjected to heating in N_2_ gas from ambient temperature to 110 °C until complete dehydration. X-ray photoelectron spectroscopy (XPS) was performed using monochromatic Al K-alpha radiation at a pressure of 10⁻^9^ mbar, with a spot size of 400 μm, an energy pass of 200 eV for the full spectrum, and a narrow spectrum of 50 eV, utilizing the K-ALPHA (Thermo Fisher Scientific, USA).

### ANN modeling

The simulation of the biological human brain networks and prediction of the complex correlations between inputs and outputs is called ANN modeling. The ANN model is a complex and flexible model built by the artificial neuron. The function of artificial neurons is to receive, store, and process information. The feed-forward back-propagation neural network (BPNN) is considered the most effective and appropriate artificial neural network (ANN) model. The BPNN consists of an input layer (IL), hidden layers (HNs), and an output layer (OL). The ANN approach is the adsorption of MB using the NDULB. The adsorption of MB using the NDULB is represented by MATLAB R2015b using the Levenberg Marquart (LM) training algorithm. The training, validation, and testing sample data ratio was 70:15:15. The optimal architecture of the ANN consisted of six hidden layers (HLs), each containing 11 hidden neurons, which achieved the highest R^2^and the lowest MSE^62^. 2–15 neurons were examined for the training stage to get the best-fit ANN approach model. The best-fit ANN is composed of four inputs and one output. The four inputs were the adsorbent dosage of the (NDULB) (mg), the PH of the MB dye, the initial concentration of the MB dye, and the contact time (min). The only output was the MB removal^63^. The figure below displays the optimal ANN architecture.

## Results and discussion

### Characterization of biochars

#### Characteristics of NDULB

**Fig. 2:**
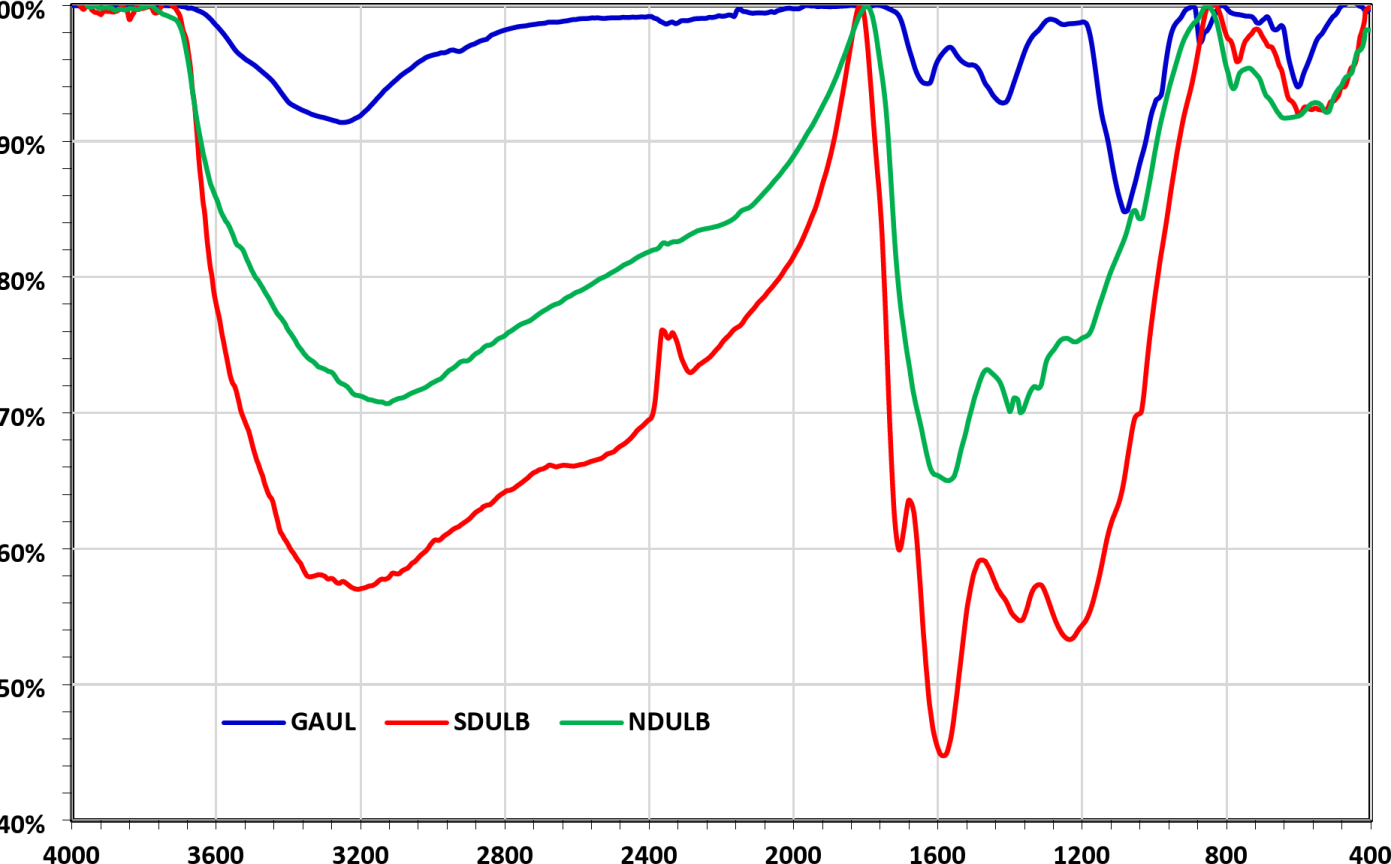
Green algae *U. lactuca* (GAUL) FTIR graphs of SDULB and NDULB.

FTIR spectroscopy was employed to identify the functional groups present on the surface of the GAUL, SDULB, and NDULB adsorbent. Figure 2 compares the FTIR spectra of NDULB and *U. lactuca*. The FTIR spectra of the materials indicate alterations in their functional groups. The broadband peaks at 3253.52 cm⁻¹ for *U. lactuca* and at 3209.79 cm⁻¹ and 3253.52 cm⁻¹ for SDULB and NDULB, respectively, correspond to the stretching vibration of the O-H group observed in all three samples. The high adsorption peak at 2926.03 cm⁻¹ indicates the presence of –CH₂ stretching groups in GAUL (Fig. 2). These groups were broadband at around 2600 cm–1 in SDULB and NDULB, as shown in Fig. 2. The small adsorption band at 1789.23 cm⁻¹ is attributed to the C = O stretching vibration of the anhydride groups in GAUL (Fig. 2). This band then vanished in NDULB at 1706.04 cm^–1^ and transformed into a carboxyl group in SDULB (Fig. 2). Nonetheless, the strength rose to 1706.04 cm^–1^when SDULB was compared to raw GAUL, indicating that the carbonyl (C = O) group may be enhanced by sulfuric acid treatment^64^. The band in GAUL at 1626.49 cm^–1^ indicates that the *β*-ketone’s C = O stretching oscillation was mostly absent in the SDULB and NDULB (Fig. 2). This oscillation, which may be a stretching vibration of –C = C– in SDULB and –C = C– or –C = N– in NDULB, progressed with high intensity to 1584.88 cm^–1^ in SDULB and 1573.06 cm^–1^ in NDULB (Fig. 2). The GAUL’ C–O functional group is represented by the peaks at 1418.32–1251.00 cm^–1^ (Fig. 2). This group was displaced to 1399.55, 1368.94, 1320.91, and 1220.42 cm^–1^, which are representative of the –NH-groups in NDULB (Fig. 2), from the band at 1370.66 and 1232.94 cm^–1^ in SDULB, which displayed the SO stretching vibration. The dehydration procedure using H_2_SO_4_ also created a broad peak encompassing the area between 768.68 and 531.57 cm^–1^. The –SO_3_H and SO groups that were produced in SDULB were the cause of these peaks. These bands demonstrate that the SDULB is created due to the GAUL treatment with H_2_SO_4_. GAUL demonstrated a more significant enhancement in the -C-O-C- asymmetric stretching group (Fig. 2) in contrast to GAUL, which had a feeble band at 1080.35 and 872.49 cm^–1^^25,65–67^. In NDULB, these bands were moved to 1038.79 (Fig. 2)^66^.

**Fig. 3:**
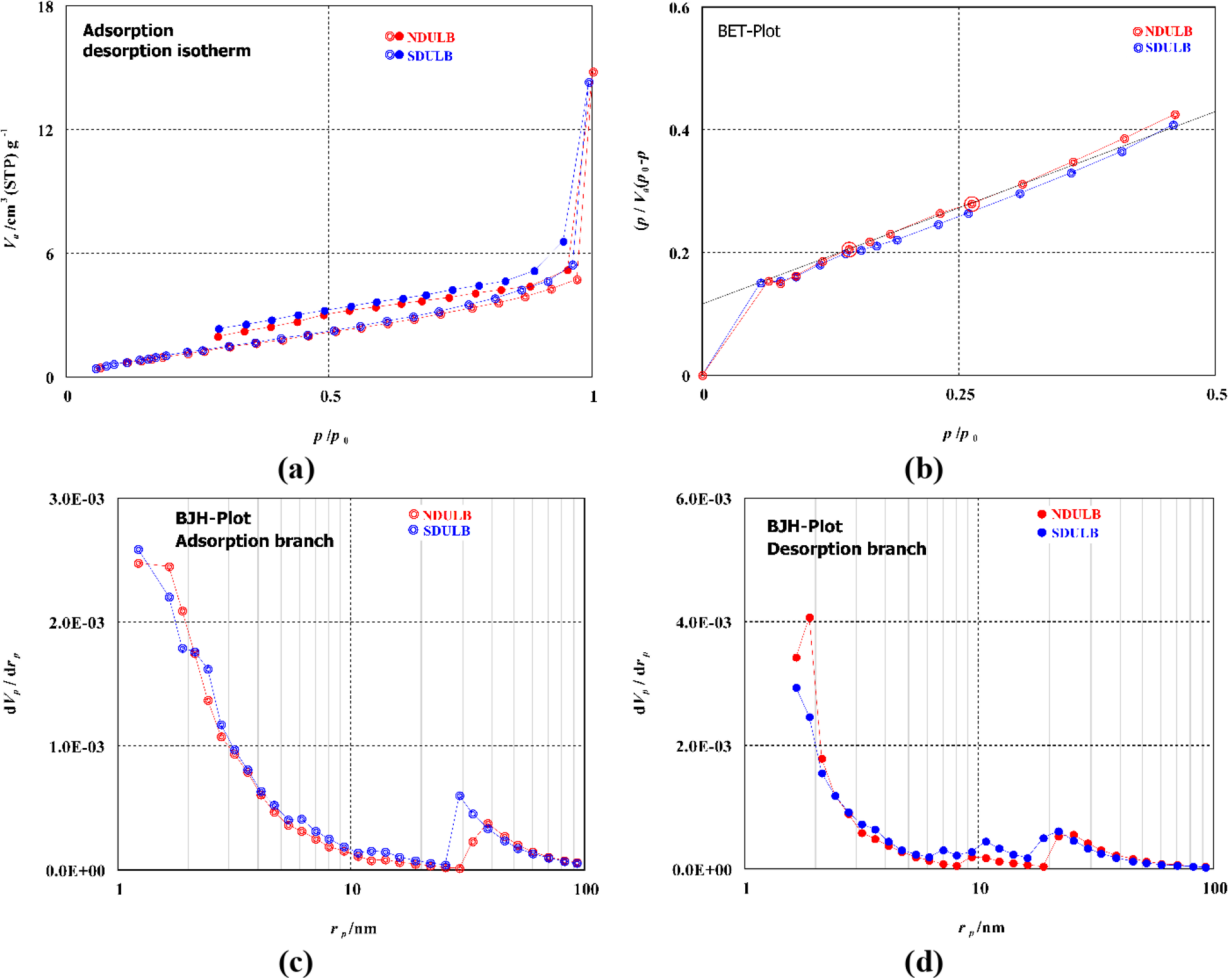
(**a**) Drawing of N_2_ Adsorption-Desorption, (**b**) Drawing of the BET, (**c**) The BJH adsorption drawing, and (**d**) The BJH desorption drawing of the SDULB (red) and NDULB (blue).

It was determined how H_2_SO_4_ changed the surface characteristics of the GAUL and how ammonia hydrothermal changed the surface properties of the SDULB by analyzing the N_2_ adsorption-desorption isotherm of the SDULB and NDULB. The mesopore and specific surface areas of SDULB and NDULB were determined using the BET and BJH methods. Figure 3 illustrates the textural characteristics of NDULB and SDULB, including the mesopore distribution peak, macro layer volume, mass of mesopores, mesopore area, average pore diameter, and total volume of pores. At 6.34 and 5.85 m^2^/g, respectively, the BET-specific surface area of the SDULB and NDULB is relatively modest. SDULB and NDULB’s monolayer volume values were 1.4567 and 1.3451 cm^3^ (STP) g^–1^, respectively. SDULB and NDULB have respective total pore volumes of 2.0309 × 10^–2^ and 1.7219 × 10^–2^ cm^3^/g. SDULB and NDULB had mean pore sizes of 12.812 and 11.764 nm, respectively. According to the SDULB adsorption investigation, the mesopore volume peak value, meso surface area peak value, and mesopore distribution peak values were 2.1338 × 10^–2^ cm^3^/g, 7.0477 m^2^/g, and 1.22 nm, respectively. The results of the NDULB adsorption investigation showed that the mesopore volume peak value, meso surface area peak value, and mesopore distribution peak values were 1.8124 × 10^–2^ cm^3^/g, 6.5913 m^2^/g, and 1.22 nm, respectively. Mesopore distribution peak value, meso surface area, and mesopore volumes were found to be 1.66 nm, 4.3323 m^2^/g, and 1.8970 × 10^–2^ cm^3^/g, respectively, in the SDULB investigation of desorption. Mesopore distribution peak value, meso surface area, and mesopore volumes were found to be 1.88 nm, 4.3782 m^2^/g, and 1.7847 × 10^–2^ cm^3^/g, respectively, in the NDULB investigation of desorption. The results for SDULB and NDULB are very close.

**Fig. 4:**
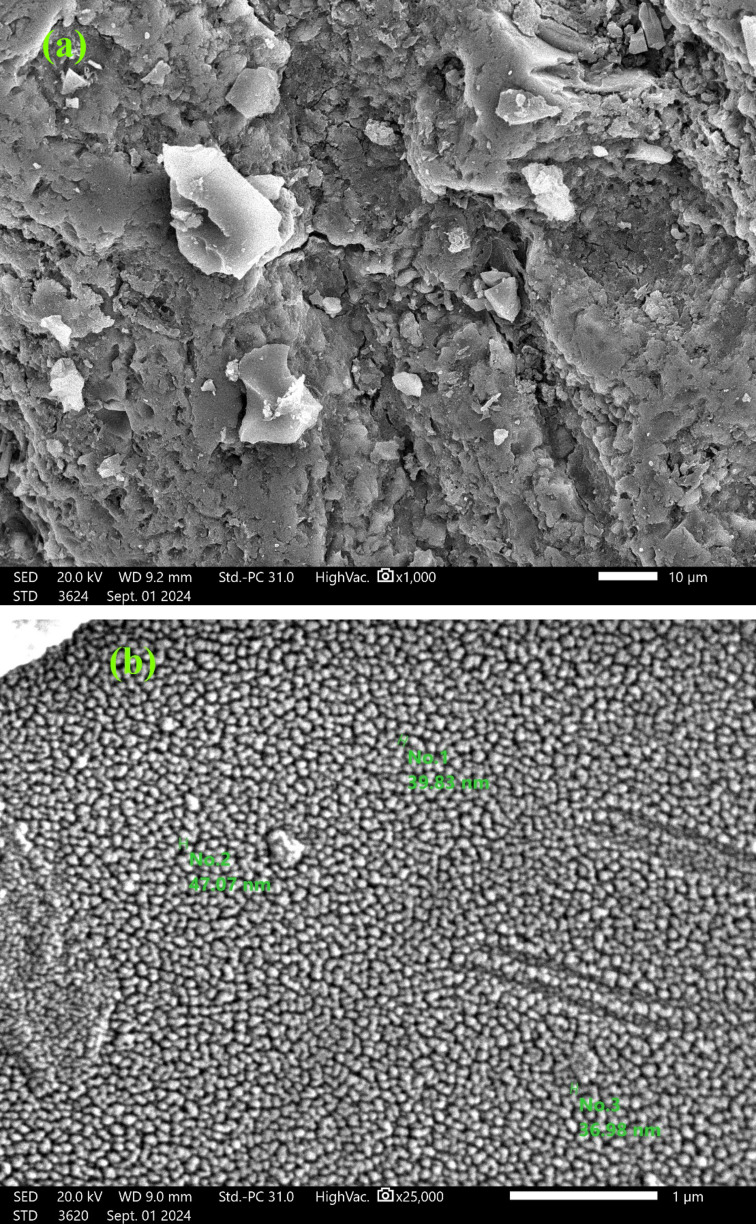
SEM image of NDULB at ×1,000 (**a**) and ×25,000 magnification (**b**) at 20.0 kV.

SEM pictures of the NDULB are shown in Fig. 4, proving its purity and absence of impurities. The SEM image in Fig. 4a indicates that the NDULB powder exhibits irregular shapes and less porose due to the presence of different nitrogen groups on the biochar surface due to the reaction with NH4OH. The SEM image in Fig. 4b shows essential information about the particle’s shape and size. The NDULB’s pore structure held up well after the intense NH_4_OH treatment. According to the particle size distribution, the average particle size distribution of the NDULB was 41 ± 7.65 nm, with the particle sizes falling between 36.98 and 47.07 nm.

The SDULB and NDULB’s chemical makeup was examined by EDX. The proportion of each SDULB and NDULB component is illustrated in Table 1. Furthermore, it reveals that the proportion of carbon mass in the SDULB and NDULB samples is 53.04 ± 0.29 and 44.96 ± 0.25%, respectively. The oxygen, sulfur, and nitrogen percentages in SDULB and NSULB are around 40.77 and 1.24%, respectively, and 36.56, 0.63, and 13.65%, respectively.

**Table 1:** The prepared GABS’s X-ray spectrometry (EDX) findings.

Elements	SDULB		NDULB	
	Mass%	Atom%	Mass%	Atom%
C	53.04 ± 0.29	61.80 ± 0.34	44.96 ± 0.21	52.46 ± 0.25
O	40.77 ± 0.56	35.66 ± 0.49	36.56 ± 0.50	32.03 ± 0.44
S	1.24 ± 0.05	0.54 ± 0.02	0.63 ± 0.03	0.27 ± 0.01
N	-----	-----	13.65 ± 0.45	13.66 ± 0.45
Si	1.24 ± 0.05	0.54 ± 0.02	0.77 ± 0.04	0.38 ± 0.02
Ca	3.21 ± 0.10	1.12 ± 0.03	3.44 ± 0.09	1.20 ± 0.03
Total	100.00	100.00	100.00	100.00

**Fig. 5:**
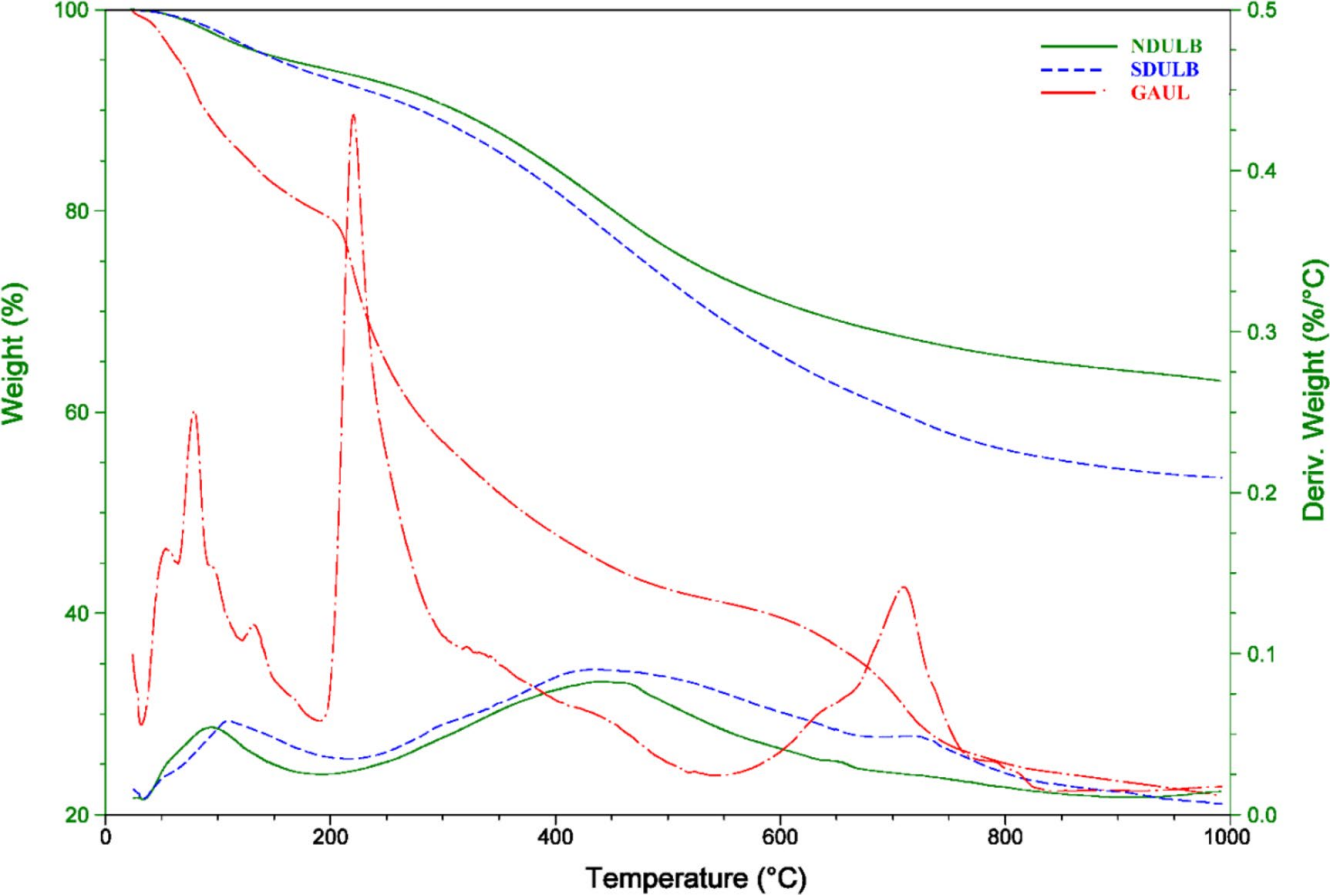
Diagrams showing TDA and TGA for GAUL, SDULB, and NDULB from 0 to 1000 °C.

The structural variations impact on the degradation behavior and operational temperature of raw Green algae *U. lactuca* (GAUL), SDULB, and NDULB samples was evaluated by TGA. The materials were subjected to cooking in a N_2_ atmosphere at temperatures between 50 and 1000 °C. Figure 5 displays the Differential Thermal Analysis (DTA) and TGA curves for the raw Green algae *U. lactuca* (GAUL), SDULB, and NDULB samples. The initial weight loss, which peaked before 220 °C, was attributed to the evaporation of water in the GAUL, SDULB, and NDULB samples. The GAUL, SDULB, and NDULB samples experienced weight loss due to the degradation of various acidic oxygen functional groups when the temperature exceeded 200 °C. With a total weight loss of 78.02%, GAUL displays three weight reductions at temperatures between 25 and 190, 190 and 545, and 545 and 1000 °C. In addition, SDULB exhibits three weight losses during 25–220, 220–690, and 690–1000 °C, for a total weight loss of 43.53%. This helps to explain why SDULB is more stable than GAUL (Fig. 5). The stability of NDULB in comparison to the GAUL and SDULB is explained by the three weight losses it exhibits at temperatures between 25 and 190, 190 and 700, and 700 and 1000 °C, for a total weight loss of 36.88% (Fig. 5). The TGA curves of the GAUL, SDULB, and NDULB samples converged at temperatures higher than 220.44, 433.27 °C, and 441.81 °C, respectively, due to the breakdown of carbon in biomass.

Figure 5 shows the DTA graph for the GAUL, SDULB, and NDULB samples. The DTA curves of SDULB and NDULB exhibited peaks at three (*T*_*f*_, 108.16, 433.27, and 725.52 °C) and two (92.02 and 441.81 °C) temperature points, respectively, whereas the raw GAUL sample showed three distinct peaks at *T*_*f*_, 78.97, 220.44, and 709.64 °C (Fig. 5). Three and two separate degradation bands were produced by dehydrating raw GAUL and then treating it with NH_4_OH at 180 °C, according to the DTA curve showing the creation of SDULB and NDULB samples from raw GAUL. The deterioration bands in the SDULB and NDULB samples appeared at a higher temperature following treatment, indicating that the described treatments significantly impacted the degree of degradation.

**Fig. 6:**
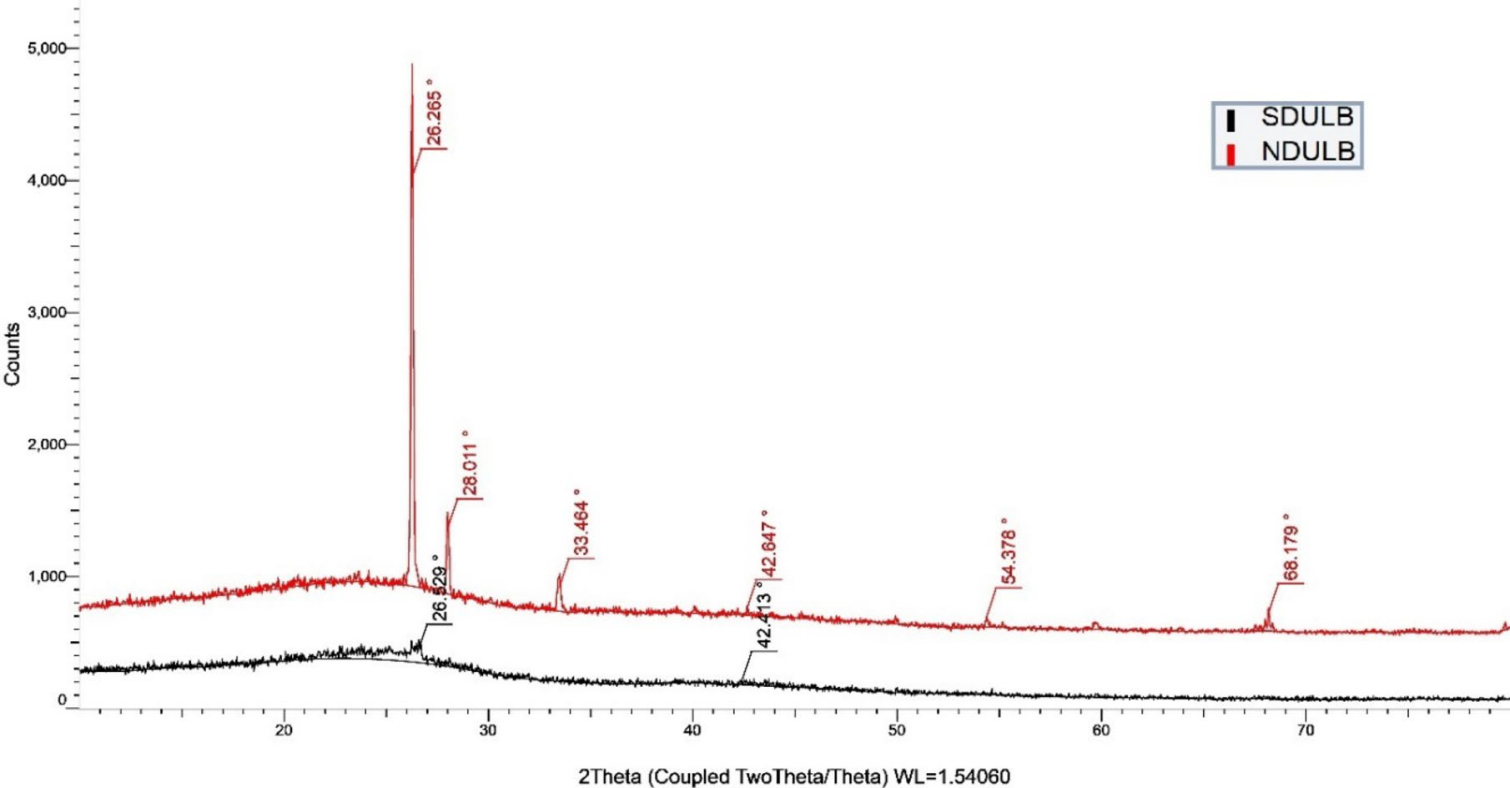
XRD diagram of synthesized SDULB and NDULB biochar.

Figure 6 presents the XRD data for SDULB and NDULB, revealing an amorphous carbon structure characterized by randomly aligned aromatic sheets. A small peak is located at 2*θ* ~ 42.4 and 42.6 for SDULB and NDULB, respectively, while a broad peak is recognized as the C (002) diffraction peak in the range of 2*θ*~ 26.6 and 26.3. Other peaks may signify several inorganic compounds, including quartz and albite (a mineral containing plagioclase feldspar)^67–69^.

**Fig. 7:**
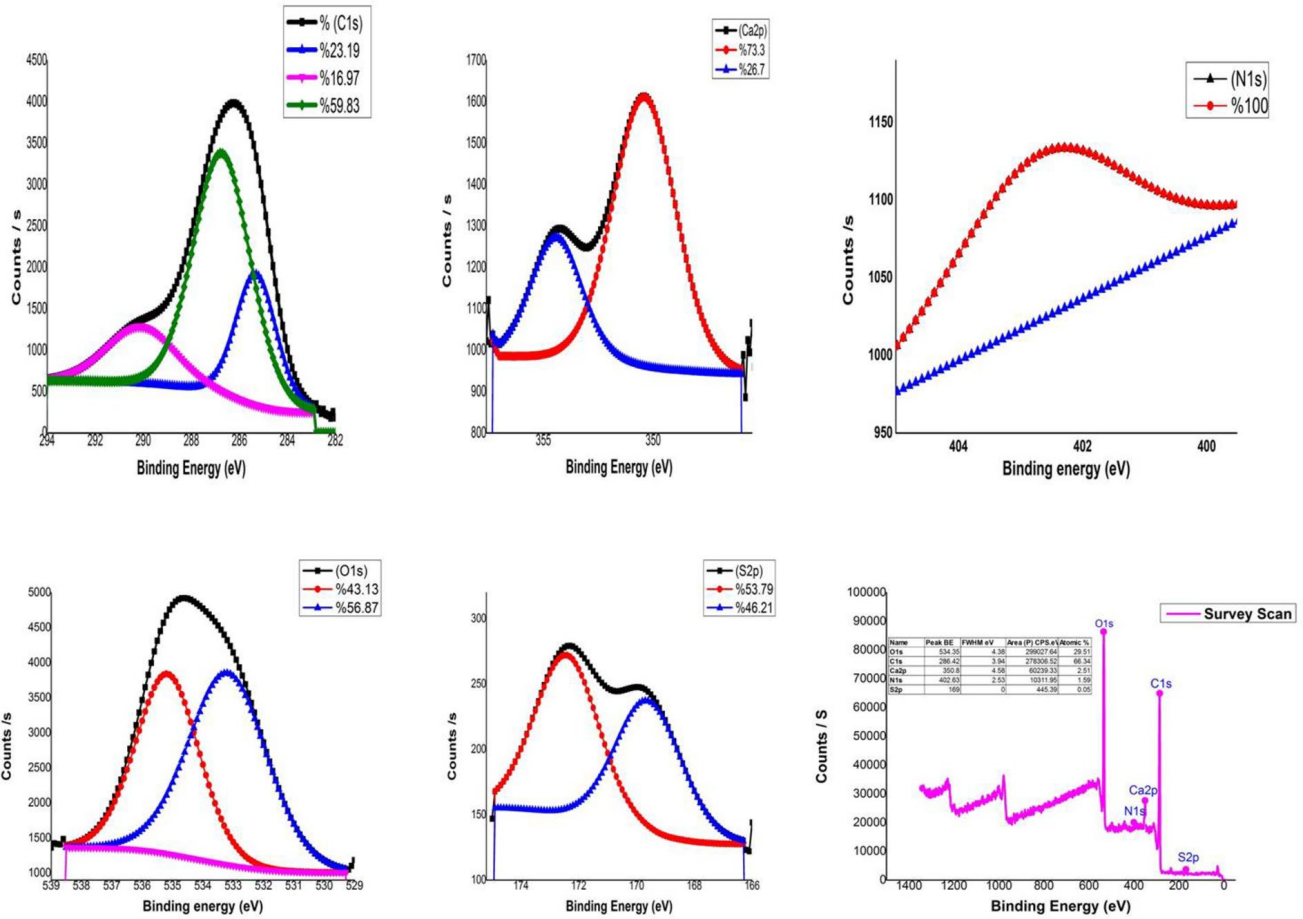
Overview of XPS spectrum of NDULB adsorbent with 1 eV resolution.

The functional groups on the nitrogen-doped Ulva lactuca Biochar (NDULB) surface were qualitatively examined using XPS^70–73^. The broad complete XPS spectra of NDULB are illustrated in Fig. 7. The graphs demonstrate that N has been effectively maintained on NDULB by showing the distinctive peaks of C1s, O1s, N1s, Ca2p, and S2p. The peaks at 286.42, 534.35, 402.63, 350.8, and 169 eV are shown in Table 2 to correspond to C1s, O1s, N1s, Ca2p, and S2p, respectively.

The C1s spectrum may be deconvoluted into three peaks centered at 285.31 (23.19%), 286.72 (59.83%), and 290.1 eV (16.97%), assigned to sp^3^-C hybridized C-C bonds, C − O−C bonds, and COOH group, respectively (Fig. 7)^70,71^. The N1s XPS spectra of NDULB show one peak at 402.58 corresponding to (NH_3_salt)^74^. The O1s XPS spectra of the NDULB display two peaks, as illustrated in Fig. 7, at 533.19 eV (56.87%), 535.17 eV (43.13) and 535.93 eV (3.98), corresponding to the OH group, C-O bonds and (H_2_O), respectively^75,76^. The Ca2p peak of NDULB is illustrated in Fig. 7, and the binding energies of 350.4 eV (73.3%) and 354.1 eV (26.7%) correspond to the peaks of Ca 2p_1/2_^77^. The S2p peak of NDULB is illustrated in Fig. 7, with two binding energies of 172.41 eV (53.79%) and 169.61 eV (46.21%) corresponding to the peaks of S2p_3/2_for sulfate species (SO₄²⁻)^78^.

**Table 2:** XPS analysis spectra of NDULB.

Name	Peak (BE)	FWHM (eV)	Area (*P*) CPS. eV	Atomic %
C1s	285.31	1.99	3422.07	23.19
	290.1	3.37	2496.54	16.97
	286.72	2.78	8820.50	59.83
Ca2p				
	350.40	3.37	2375.79	73.30
	354.41	2.73	862.84	26.70
N1s				
	402.58	3.59	389.58	100.00
O1s				
	535.17	2.45	6792.87	43.13
	533.19	3.03	8970.82	56.87
S2p	172.41	2.64	349.84	53.79
	169.61	2.71	301.07	46.21

### Adsorption of MB dye using NDULB

#### Effect of initial pH

**Fig. 8:**
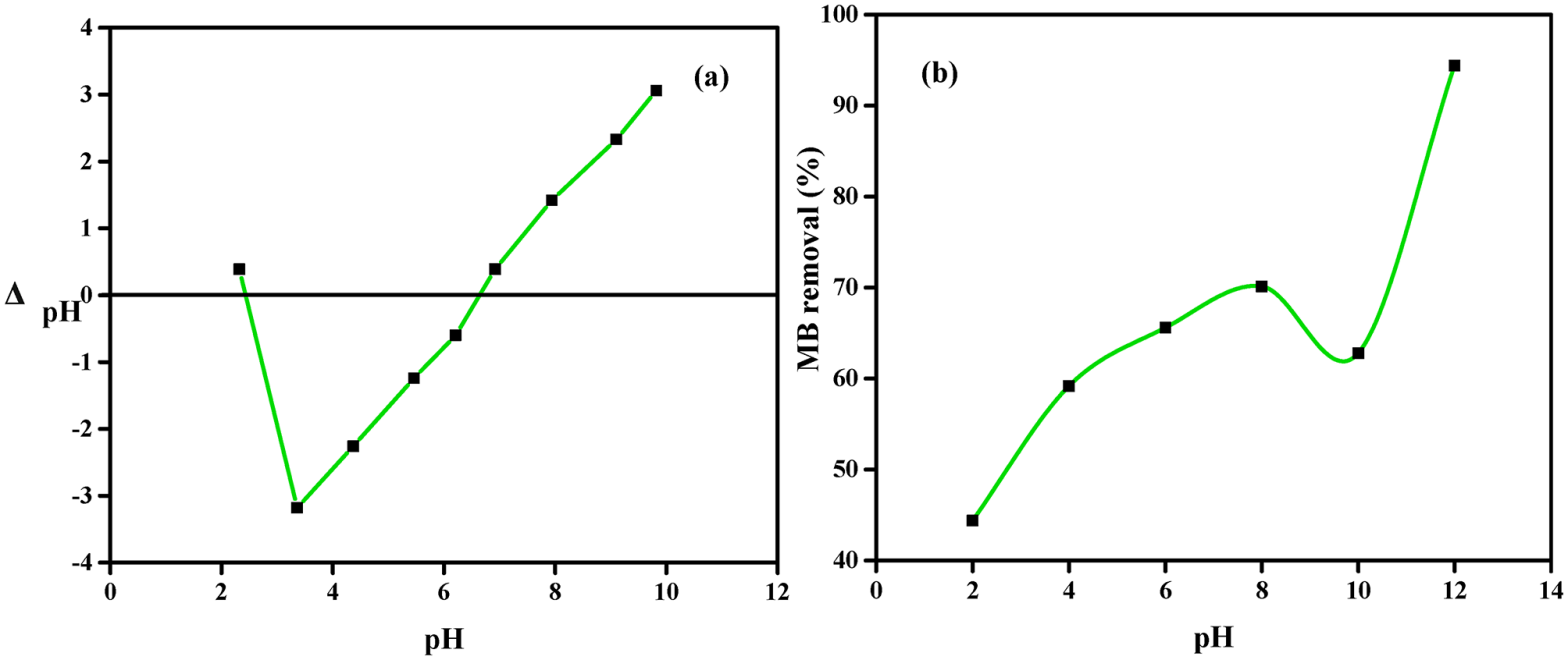
Sorption of MB dye onto NDULB (**a**) zero point charge and (**b**) pH on the removal % of MB dye (MB dye = 100 ppm, adsorbent = 1.0 g L^−1^, temperature = 25 °C).

The initial pH of the effluent from the textile industry exhibits significantly. The initial pH of the solution has an effect on the carboxyl, amino, and hydroxyl groups on the surface of NDULB, which in turn has an impact on the adsorption process^9^. A set of conditions, including a starting MB dye concentration of 100 ppm, a contact time of 30 min, an adsorbent dosage of 1.0 g L^−1^, and a temperature of 25 °C, were used to adjust the pH of the MB dye solution and study the mechanism of MB dye removal onto NDULB. The point of zero-charge (pH_PZC_), based on the result displayed in Fig. 8a, was calculated to be 6.95. When the solution’s pH was higher than pH_PZC_, the active sites on the biosorbent surface were negatively charged and positively charged when it was after or below the pH_PZC_. Since the MB dye has a positive charge in the solution, it is categorized as cationic^79,80^.

Reduced MB dye adsorption occurs at lower pH levels, such as pH 2, because of electrostatic repulsion between the adsorbent and the dye molecules caused by the positive charge on the adsorbent’s surface. Conversely, the deprotonation of acidic functional groups causes the surface of NDULB to become more negatively charged when the pH rises over the isoelectric point (pH 12 in this case). The adsorbent and positively charged methylene blue dye molecules are attracted to each other electrostatically, which makes it easier for the MB dye to adhere to the NDULB surface. Electrostatic repulsion is lessened when the pH rises from a low value (such as pH 2) because the adsorbent surface now has a negative charge instead of a positive one. Elect electrostatic attraction eventually results when the pH climbs above the isoelectric point (6.95). As a result, the adsorption capacity of methylene blue on NDULB improved with increasing pH because of the very negative surface charge of NDULB, which facilitates the removal of the cationic dye molecules. Ideal levels are reached when the pH is much over the isoelectric point. This pattern is consistent with the ideas of electrostatic interactions and surface charge in the context of removal processes. Shoaib et al. observed a similar trend in their investigation of methylene blue dye removal^81^.

Several important conclusions can be made from the data analysis of the investigation on the effectiveness of MB dye removal utilizing NDULB under various pH circumstances (Fig. 8b). The overall pattern shows that, except for pH 10, the adsorption effectiveness of methylene blue dye by NDULB progressively improved as the pH increased from 2 to 12. This suggests that throughout the pH range of 2 to 8, which is slightly acidic to neutral, NDULB has a reasonably constant adsorption capacity. The greatest MB dye removal (94.4%) by NDULB was achieved at pH 12. The MB dye removal change graph shows that when pH was raised from 2 to 8, MB dye removal rose from 44.4 to 70.1%. There is a minor drop when the pH is raised from 8 to 10, followed by a dramatic increase when the pH is raised from 10 to 12. Jabar et al.^64^found that when the pH was raised from 1 to 9, the elimination of crystal violet (CV) dye continuously increased the amount of adsorption. After that, there was a slight decline^64,82^. In low pH aqueous media, methylene blue molecules ionized in water, producing cations that competed with protons (H^+^) for the accessible anionic functional groups on the surface of NDULB. As the pH increased from 2 to 8, the aqueous solution’s protonation relaxed, and the quantity of methylene blue adsorbed increased (Fig. 8b). The rise in the fraction of MB dye adsorbed and its uptake, as the pH value increased from 2 to 8, may be attributed to an enhancement in the electrostatic attraction between the cationic dye and the anionic surface charge of the NDULB adsorbent. When Razzak et al.^83^investigated the removal of the methylene blue dye using a bio-adsorbent generated from Schinus molle, they discovered that increasing the solution’s pH from 2 to 8 increased the percentage of adsorption removal from 30 to 100%^83^. The literature has numerous reports of comparable results for removing methylene blue dye^84,85^. 

The hydroxyl (-OH), amino (-NH_2_), and carboxyl (-COOH) functional groups on the biochar surface create an attractive/repulsive interaction between the adsorbate and the adsorbent, which makes them very sensitive to the pH of the effluent. The aqueous medium becomes ionized at low pH levels, depositing H_3_O^+^ on the NDULB surface-active sites to positively charge the surface. Electrostatic repulsion shields the cationic-charged NDULB surface from cationic-charged MB dye molecules. Consequently, dye elimination is limited. As the pH of the solution rises, the active sites on the NDULB surface become less protonated, which facilitates the migration of MB dye from the aqueous solution to the NDULB surface. The improvement in adsorption efficacy with raising pH could be attributed to a reduction in competition between H_3_O^+^ ions and the methylene blue cationic MB dye molecules for adsorption on the adsorbent’s active sites. Moreover, the re-emergence of -CO-, -NH-, and -OH functional groups with increasing pH may have led to an evolution in the electrostatic interaction between anionic NDULB active sites and cationic methylene blue molecules, which might have contributed to the increase in adsorption effectiveness.

#### Impact of contact time

**Fig. 9:**
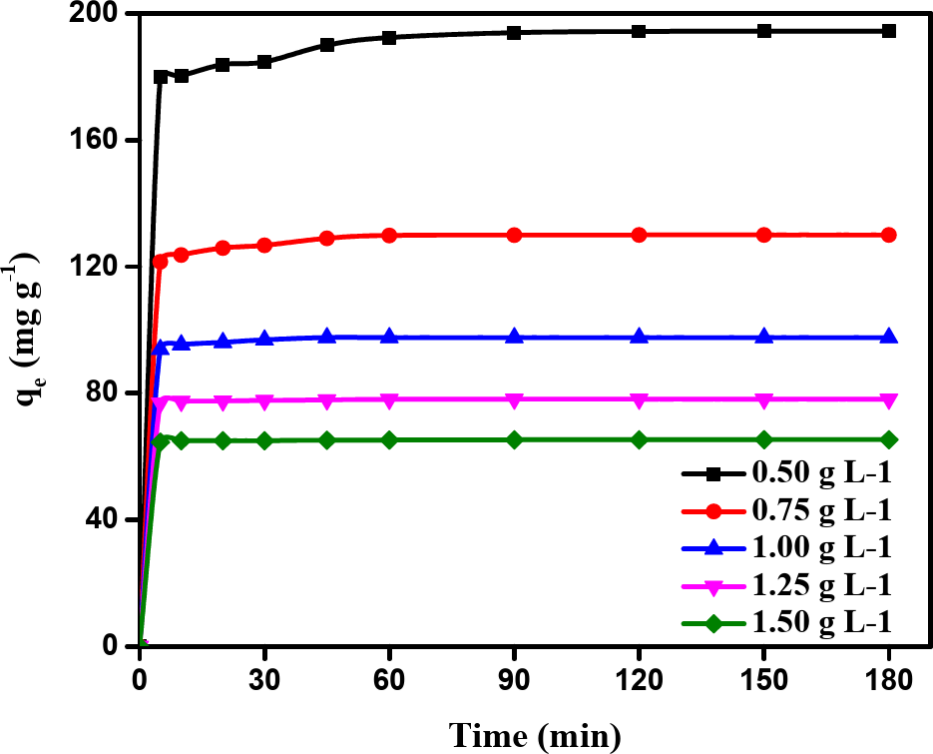
The removal of MB dye by NDULB (MB dye = (100–200 ppm), doses (0.50–1.50 g L^−1^), and Temperature = 25 °C).

Datasets analysis of NDULB’s methylene blue dye removal capacity across various adsorbent concentrations over time revealed many relevant details about the equilibrium states and adsorption kinetics. The data in Fig. 9 show how quickly NDULB absorbed MB dye over time, suggesting a typical first stage of adsorption typified by many accessible active sites. Adsorbent concentration significantly impacts adsorption capacity; at lower concentrations, the removal capacity of methylene blue rises quickly, reaching 179.87 mg g^–1^ in 5 min and rising to 194.34 mg g^–1^ at 180 min. This implies that the ratio of accessible adsorption sites to methylene blue dye is large at lower adsorbent concentrations, allowing for better saturation of each adsorbent. Nonetheless, the initial adsorption capacity falls with increasing NDULB dosage, suggesting a dilution impact or less accessible adsorption sites per unit of adsorbent as a result of aggregation or decreased surface area exposure. The adsorption capacity trend does not adhere to a linear relationship as the NDULB dose rises, indicating declining returns with rising concentration. This could be because aggregates form at higher concentrations and adsorption sites overlap, reducing the effective surface area available for the removal.

The adsorption capacity of methylene blue by NDULB rose gradually with increasing NDULB dose (0.5–0.75 g L^–1^) and achieved saturation in 60–120 min. Additionally, when NDULB concentrations are 1.0 to 1.5 g L^–1^, saturation is reached in 10 min; for a dose of 0.5 g L^–1^ NDULB, this time is 120 min with an adsorption capacity of 194 mg g^–1^. The observed rise in the adsorption capacity of methylene blue dye by NDULB with time, achieving saturation after differing times at various NDULB doses, was also in line with the results of several relevant studies. For example, one study indicated that activated carbon treated with surfactants significantly enhances the adsorption efficacy of cationic dyes, including MB dye^86^. This outcome provides information on how cationic dyes are removed and aligns with the research results on removing methylene blue and its complexes with montmorillonite^87^. The investigation provided more understanding of the removal behavior of cationic dyes by demonstrating the selective adsorption of cationic dyes^65^. Additionally, Yamaguchi et al.‘s^66^ studies of removing the cationic dye methylene blue dye shed light on the behavior of cationic dyes during adsorption.

The rapid attainment of saturation with an increase in the adsorbent material (NDULB) ratio and the subsequent decline in methylene blue dye adsorption capacity can be ascribed to numerous critical aspects associated with the removal process and the physical characteristics of the system. First, the available surface area for adsorption determines how well the adsorbate (Methylene Blue dye) interacts with the adsorbent (NDULB). The percentage of adsorbent material is raised to improve the accessible surface area for the removal process. Nonetheless, there is a limit to how much methylene blue dye may be absorbed into the surface area. The adsorption capacity is not increased by accumulations in the adsorbent material beyond a certain point.

Second, when additional adsorbent material is added, there is greater competition among the adsorbent particles for the limited number of adsorption sites. The MB dye molecules compete with one another to establish bonds with the few active sites on the adsorbent surface, resulting in a higher rate of saturation and a lower overall adsorption capacity. Thirdly, the diffusion period of MB dye molecules into the active regions on the adsorbent surface may be prolonged in some situations by the addition of additional adsorbent material. Because extended diffusion paths slow down the pace at which methylene blue dye molecules may bind and pass through the adsorbent, they may hasten the saturation of adsorption sites. Finally, the system may approach the point at which mass transfer constraints become significant as the proportion of NDULB material rises. This indicates that the rate at which methylene blue molecules move from the bulk solution to the NDULB surface will be a limiting factor. As a result, the adsorption capacity saturates faster when the adsorption rate is less than the rate at which the molecules of methylene blue dye reach the adsorbent surface^32,67^.

#### Impact of initial MB dye concentration

**Fig. 10:**
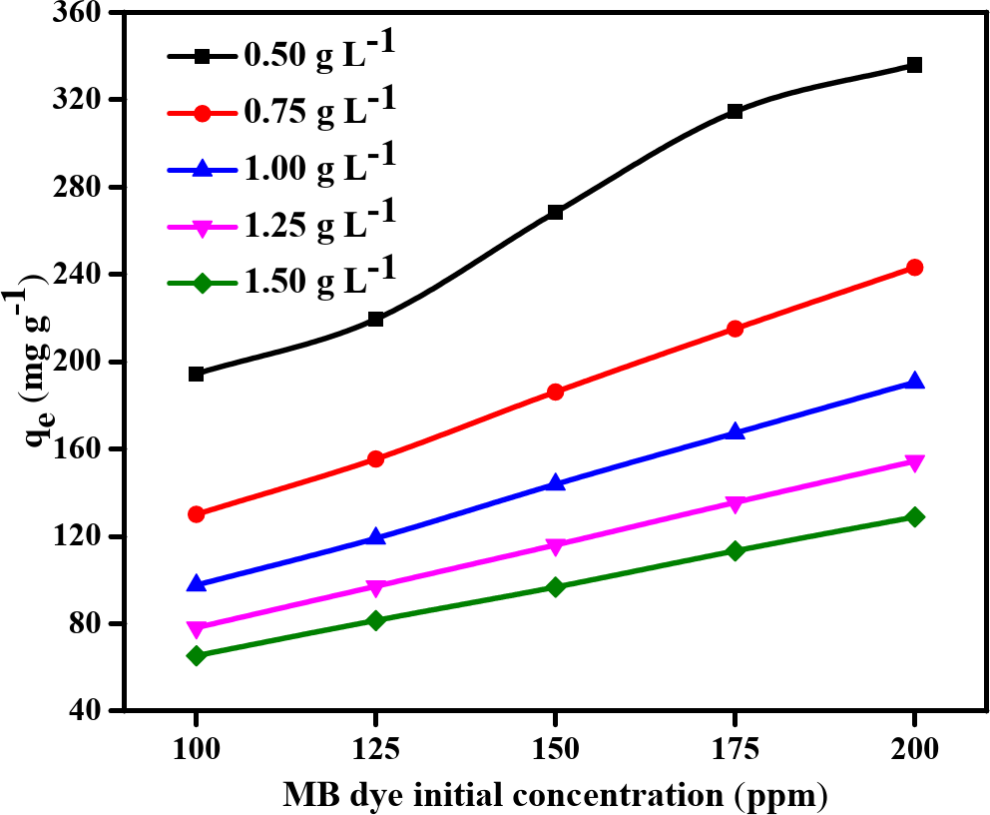
The MB dye starting concentration (100–200 ppm) impact using NDULB doses (0.50–1.50 g L^−1^) on *q*_*e*_ (mg g^−1^) and temperature (25 °C).

The graph illustrated in Fig. 10 sheds light on how the methylene blue dye’s initial concentration and ability to bind to NDULB at different adsorbent dosages relate to one another. This investigation provides important insight into how the adsorption efficiency of MB dye for varying *C*_0_ is affected by the NDULB dose. Notably, the adsorption capacity of NDULB at 0.5 g L^–1^ reached 194.34 mg g^–1^ at a starting methylene blue dye concentration of 100 ppm, demonstrating the high effectiveness of MB dye removal per unit of adsorbent. Because more active sites are accessible at the lower concentration of methylene blue dye, maximum interaction between NDULB and methylene blue dye molecules is facilitated, which accounts for this remarkable adsorption capacity. However, the adsorption capacity of NDULB at 0.5 g L^–1^ rose as well, peaking at 335.82 mg g^–1^ using 200 mg g^–1^ of MB dye as the *C*_0_ of the dye increased to 125, 150, 175, and 200 ppm. This pattern implies that a greater *C*_0_ of MB dye can strengthen the mass transfer driving force, overcoming adsorption resistance and producing a higher adsorption capacity. On the other hand, it has been determined that adsorption capacities generally decrease due to increasing adsorbent dosage in NDULB dosages ranging from 0.75 to 1.5 g L^–1^. The adsorption capacity dropped from 243.12 mg g^–1^ at 0.75 g L^–1^ to 128.82 mg g^–1^ using 1.5 g L^–1^, with an initial methylene blue dye concentration of 200 ppm.

Numerous pertinent research back up the assertion that sorbent particle aggregation at higher dosages decreases the number of active sites and effective surface area available for MB dye removal. Aggregation and interlocking of sorbent particles, which lowers the total surface area accessible for dye adsorption, are the primary causes of the observed reduction in adsorption capacity with larger sorbent dosages. Adsorbent particles may also agglomerate with higher masses, which might result in a decrease in surface area and an increase in diffusional route length^67^. The adsorption capacities at higher doses plateau as the initial methylene blue concentration increases, indicating the approach to the adsorbent’s equilibrium capacity^68^. These data support the hypothesis that, at greater doses of adsorbent particle aggregation, fewer active sites and effective surface area may be available for methylene blue removal, reducing adsorption capacity. The statistics emphasize how important the adsorbent dosage is in determining how well NDULB adsorbs. Because of their reduced overall adsorption capacity, lesser doses of NDULB may not be suitable for treating higher concentrations of methylene blue, even though they are more effective in adsorbing the dye per gram. On the other hand, larger dosages of NDULB may be needed to treat higher concentrations or larger volumes of methylene blue dye, notwithstanding their decreased effectiveness per unit mass.

#### Adsorbent dosage impact on MB dye adsorption

**Fig. 11:**
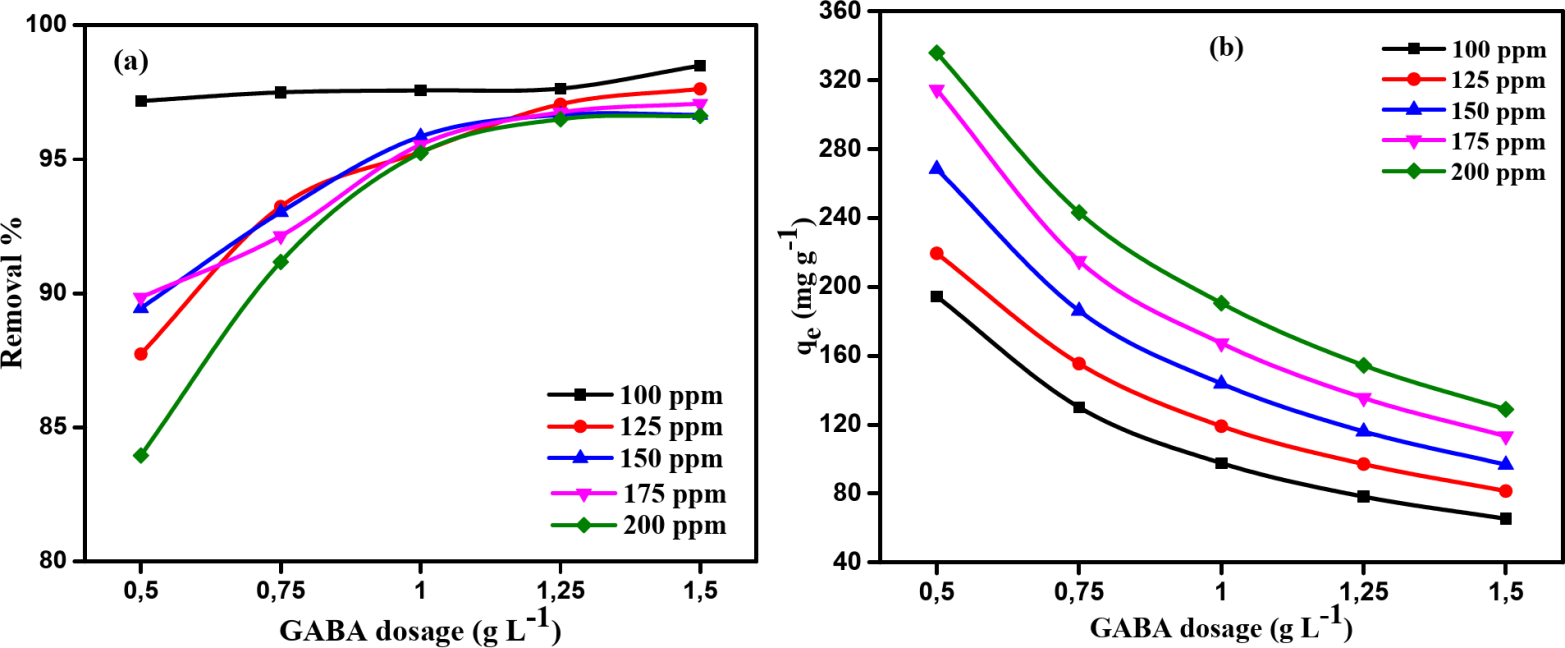
The impact of NDULB various doses (0.50–1.50 g L^–1^) of different starting MB dye concentrations (100–200 ppm) (**a**) on removal %; (**b**) on *q*_e_ (mg g^−1^), and temperature (25 °C).

The effect of an adsorbent dose on methylene blue (MB) removal is significant, as it directly influences the adsorption capacity and the efficiency of dye removal. The impact of adsorbent dosage on MB dye removal onto NDULB was investigated by varying the adsorbent dose from 0.50 to 1.50 g L^−1^, using an initial methylene blue dye concentration (100–200 ppm), a contact duration of 180 min, and a solution temperature of 25 °C, with an initial pH of 8.0. The findings are shown in Fig. 11a, b. As the adsorbent dose increases, more active sites are available for adsorption, leading to a higher percentage of dye removal (Fig. 11a). However, beyond a certain point, the equilibrium adsorption capacity (*q*_*e*_) decreases with increasing adsorbent dose (Fig. 11b). This occurs because at higher doses, the surface area becomes saturated with adsorbed dye molecules, and the excess adsorbent may lead to agglomeration, reducing the effective surface area available for further adsorption. Therefore, there is an optimal adsorbent dose that maximizes both the dye removal efficiency and the adsorption capacity, balancing the availability of active sites and minimizing the loss of adsorption effectiveness. Consequently, 84–98% of the dye was removed. For starting MB dye concentrations of 100, 125, 150, 175, and 200 ppm, respectively, the quantity of NDULB adsorbent was increased from 0.50 to 1.5 g L^–1^. As a result, the amount of methylene blue dye adsorbed at equilibrium (*q*_e_) decreased from 194.34 to 65.30, 219.34 to 81.35, 268.34 to 96.65, 314.47 to 113.25, and 335.81 to 128.82 mg g^–1^. It was found that the lowest adsorption quantity at equilibrium (*q*_e_) was achieved by an NDULB dose of 1.5 g L^–1^.

### Adsorption isotherms

**Fig. 12:**
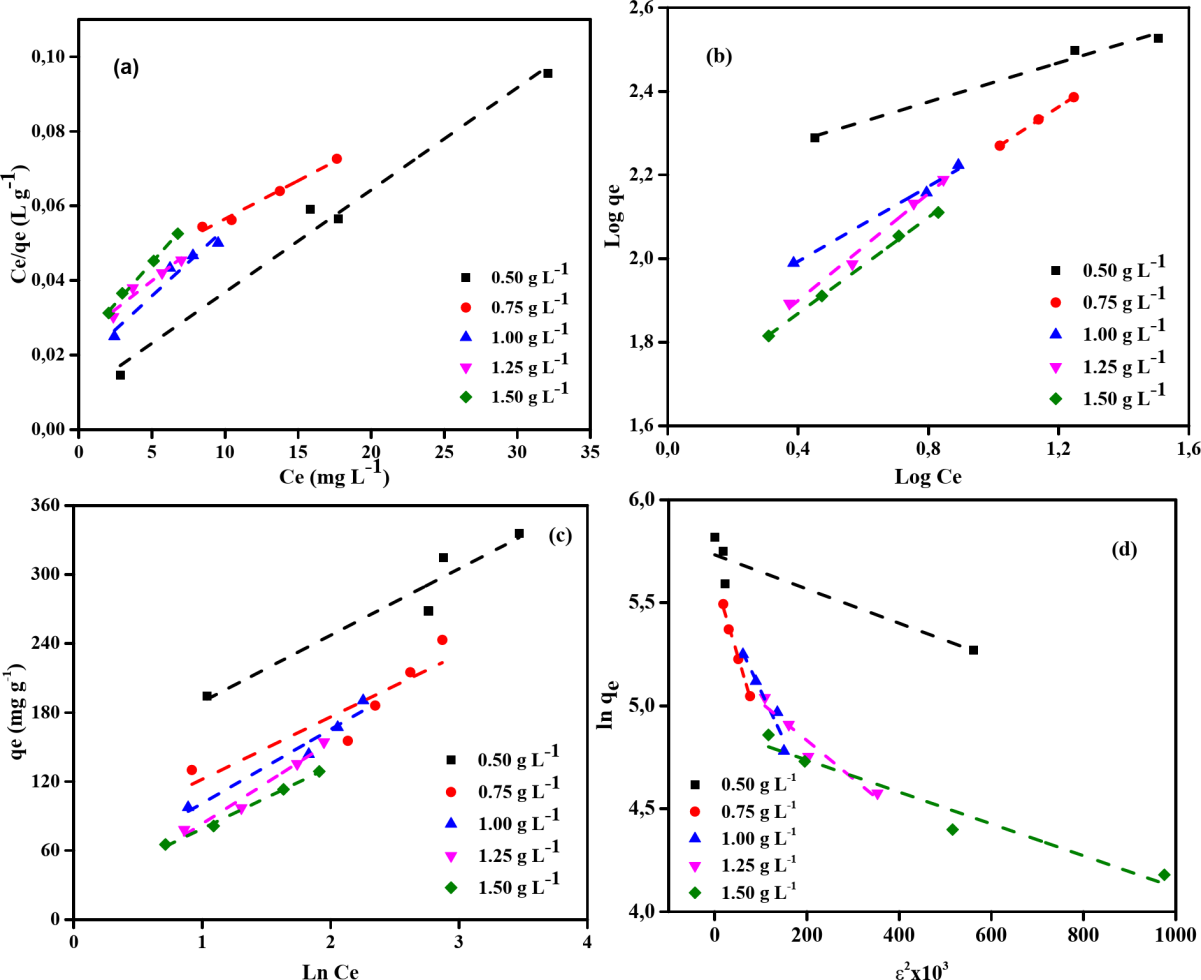
(**a**) Linearized LIM (**b**) FIM (**c**) TIM (**d**) DRIM isotherm profiles for MB dyes of initial concentration (100–200 ppm) on NDULB doses (0.50–1.50 g L^−1^) and Temperature (25 °C).

The study shown in Table 3 describes how several isotherm models are applied to explain how MB dye adsorbs on NDULB at varied concentrations (Fig. 12). Isotherm models are vital for constructing adsorption systems and for comprehending the relationship between adsorbates and adsorbents.

#### Langmuir isotherm model (LIM)

The maximum adsorption capacity (*Q*_m_) shows that smaller dosages of NDULB have a greater ability to adsorb MB dye since it declines with increasing NDULB dose (except 0.5 and 1.0 g L^–1^). The strength of the adsorption bond is reflected in the adsorption constant (*K*_L_), which changes with different NDULB dosages. The *R*^*2*^ values are between 0.943 and 0.997 for all NDULB dosages, indicating a well fit with the Langmuir model, which posits monolayer adsorption on a homogenous surface devoid of interactions among adsorbed molecules.

#### Freundlich isotherm model (FIM)

The surface heterogeneity or adsorption intensity can be inferred from the heterogeneity factor (1/*n*). Reduced values indicate a more uniform adsorption intensity. As the NDULB dose is increased, the Freundlich adsorption capacity (*K*_*F*_) falls (except 1.0 g L^–1^), suggesting lower adsorption effectiveness at larger adsorbent doses. The Freundlich model matches all concentrations well based on the *R*^2^ values.

#### Tempkin isotherm model

A possible decrease in adsorption capacity is indicated by the noticeable fall in the Tempkin isotherm constant (*A*_*T*_) with increasing NDULB dose (except 1.25 g L^–1^). Variations in the Temkin constant (*B*_*T*_) with NDULB concentration suggest changes in surface coverage and heat of adsorption. Aside from 0.75 g L^–1^, where the fit is less than ideal, the *R*^*2*^ values demonstrate that the Tempkin model fits data quite well overall.

#### Dubinin-Radushkevich (D-R) isotherm model

As the NDULB dose is increased, the theoretical saturation capacity (*Q*_m_) falls. Variations in the D-R constant (*K*), which indicate variations in adsorption energy, provide insight into the nature of the adsorption process. The kind of adsorption can be inferred from the mean free energy (*E*) of adsorption; energies below eight kJ mol^–1^ often indicate physical adsorption. The D-R model appears to fit most concentrations quite well, according to the *R*^*2*^ values, albeit it does not fit as well at 0.5 g L^–1^.

The Freundlich model best describes MB dye removal by NDULB, suggesting a heterogeneous surface with varying adsorption energies. However, the applicability of the Langmuir isotherm model (LIM) at most doses indicates that some adsorption also occurs on a homogeneous monolayer surface. The Dubinin-Radushkevich (D-R) model suggests a predominantly physical adsorption process, while the Tempkin model shows that adsorption energy decreases as NDULB concentration increases. These findings highlight the need to consider multiple isotherm models for a comprehensive understanding of the adsorption mechanism. Since LIM and Tempkin models are relevant at different NDULB dosages, the adsorption process is not entirely homogeneous or purely monolayer. The results emphasize the importance of selecting the best-fit isotherm model to predict adsorption behavior and optimize system design, particularly for scaling up from laboratory to industrial applications^88,89^.

**Table 3:** Isotherm study data of MB dye adsorption onto NDULB (MB dye = 100–200 ppm), adsorbent doses (0.50–1.50 g L^–1^), and Temperature (25 °C)).

Isotherm Model	Parameters	NDULB doses (g L^–1^)				
		0.50	0.75	1.00	1.25	1.50
LIM	*Q*_*m*_ (mg g^−1^)	370.37	476.19	277.78	322.58	227.27
	*K*_*L*_	0.29	0.06	0.20	0.13	0.19
	*R* ^*2*^	0.985	0.986	0.954	0.943	0.997
FIM	*Q*_*m*_ (mg g^−1^)	476.40	414.62	375.58	966.31	412.16
	*1/n*	0.23	0.51	0.45	0.64	0.57
	*K*_*F*_ (mg^1−1/n^ L^1/n^ g^–1^)	153.92	56.14	65.15	43.99	43.50
	*R* ^*2*^	0.984	1.000	0.992	0.991	0.998
TIM	*A*_*T*_	9.74	3.49	1.77	1.19	1.60
	*B*_*T*_	57.81	54.23	64.29	71.16	53.82
	*R* ^*2*^	0.930	0.824	0.956	0.973	0.996
DRIM	*Q*_*m*_ (mol kg^–1^)	308.80	274.93	256.60	181.25	133.02
	*K × 10*^*6*^ (mol kJ^–1^)^2^	0.80	7.50	4.80	1.80	0.80
	*E* (kJ mol^–1^)	0.791	0.258	0.323	0.527	0.791
	*R* ^*2*^	0.872	0.994	0.939	0.937	0.952

### Finding the best-fit isotherm model using error function analysis

The best appropriate model for MB dye removal on NDULB adsorbent was identified by comparing the correlation coefficients (*R*^2^) for each of the four isotherm models with the experimental equilibrium data. Another method to select the best isotherm model based on experimental data is to evaluate the values of various error functions. The following are the main functions that are used to calculate the error distribution between the estimated isotherm models and the equilibrium values: Error metrics include measures such as chi-squared error (X^2^), hybrid error function (HYBRID), Marquardt’s percent standard deviation (MPSD), sum of the errors squared (ERRSQ), average percent errors (APE), average relative error (ARE), sum of absolute errors (EABS), and others^90^. According to Table 4, the most suitable model is the Linear-LIM, as indicated by the error function terms.

**Table 4:** Some error function values of the experimental data on the MB dye adsorption by NDULB.

Isotherm Model	APE (%)	X^2^	Hybrid	ERRSQ	MPSD	ARE	EABS	RMS
Linear-LIM	0.013	0.021	0.117	3.553	0.059	0.013	8.430	0.056
FIM	0.225	4.683	29.269	745.804	1.010	0.225	115.864	1.044
TIM	0.649	75.050	395.001	14460.797	3.124	0.649	551.069	2.972
DRIM	0.636	55.383	307.682	9483.332	2.997	0.636	435.507	2.844

### Adsorption kinetic studies

**Fig. 13:**
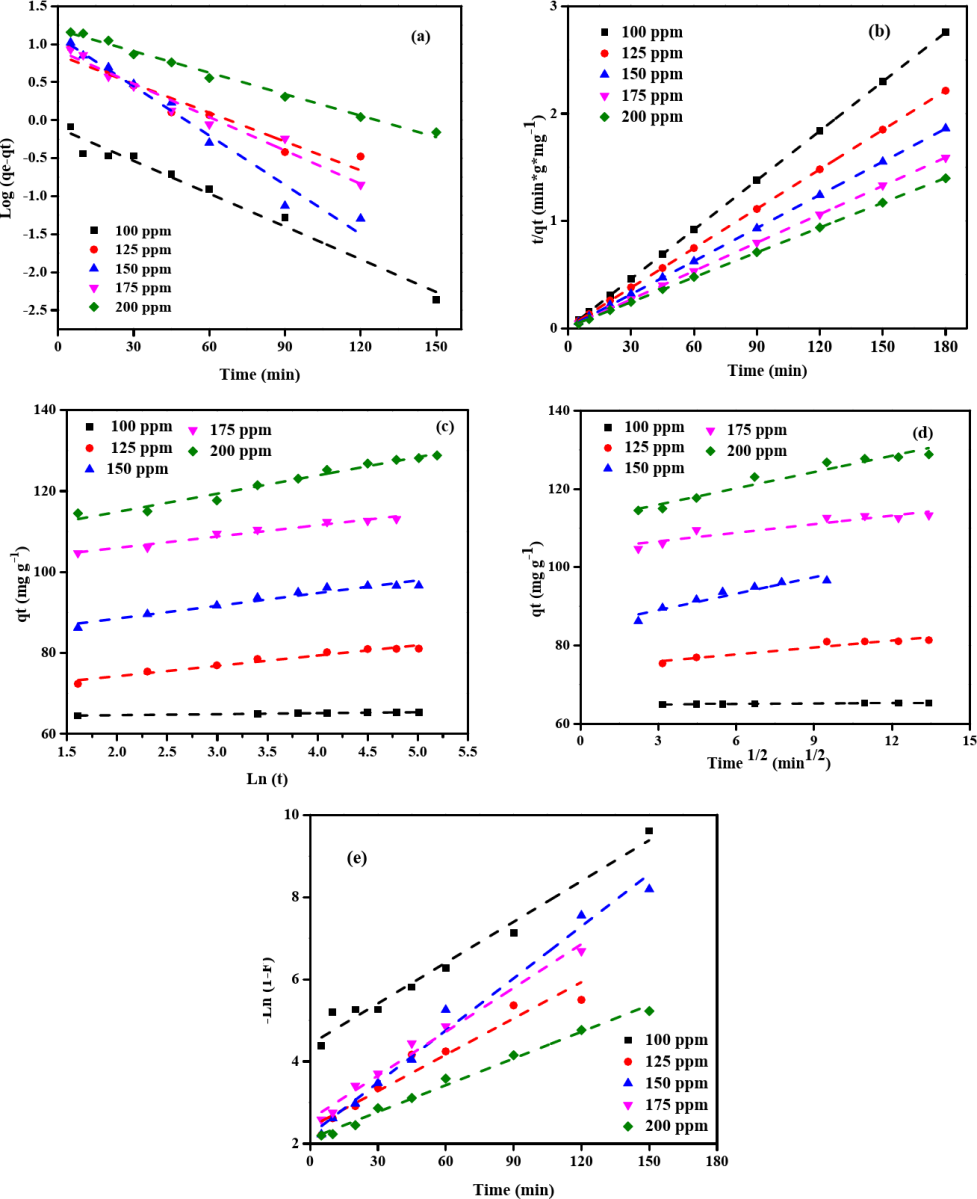
(**a**) PFOM, (**b**) PSOM, (**c**) EM, **(d**) IPDM, and (**e**) FDM kinetic models of adsorption of MB dye by NDULB adsorbent [starting MB dye concentration (100–200 ppm), NDULB dose (1.5 g L^−1^), and Temperature (25 °C)].

The kinetics of methylene blue removal on NDULB are shown in Tables 5 and 6, and Fig. 13. These kinetic models include PFOM, PSOM, EM, IPDM, and FDM. These models are necessary to understand the rate of adsorption and to choose the best model for adsorption system design and behavior prediction in real-world applications.

#### Pseudo-first-order model (PFOM)

The *q*_e, calc.,_ and *k*_1_ are compared with the *q*_e, exp_. in the PFOM (Fig. 13a). According to the PFOM, the occupancy rate of occupied adsorption sites determines the proportion of occupied to empty sites. The values of *k*_1_ depend on the starting concentration of methylene blue and the NDULB dose. For instance, the *k*_1_ value is 46.98 × 10^–3^ with a qe(calc.) of 27.33 mg g^–1^ at an initial methylene blue concentration of 100 ppm and an NDULB dose of 0.5 g L^–1^, which is lower than the experimental result of 194.34 mg g^–1^, indicating some model variance. The PFOM’s *R*^*2*^ values are also provided (Table 5), demonstrating the model’s goodness of fit with the experiments. All data sets have *R*^*2*^ reasonably high values, although some have values that are less than 0.95, indicating a less precise model fit in some circumstances.

#### Pseudo-second-order model (PSOM)

The Pseudo-Second-Order Model (PSOM) provides a better fit for MB dye adsorption onto NDULB, as it assumes that adsorption depends on both the availability of dye molecules and active sites (Fig. 13b). At most NDULB concentrations, the calculated equilibrium adsorption capacity (qe) closely matches the experimental data, with consistently high R² values, indicating a strong correlation. The initial adsorption rate (h) increases at lower NDULB concentrations, especially when MB dye concentrations are high, suggesting more efficient adsorption under these conditions. The PSOM aligns better with the experimental data compared to the Pseudo-First-Order Model (PFOM), particularly at lower NDULB concentrations where R² values approach 1.000. While PSOM provides a good fit across all MB dye concentrations, PFOM shows reasonable agreement only at certain NDULB doses, such as 0.5 g L⁻¹. These findings confirm that MB dye adsorption onto NDULB follows second-order kinetics, making PSOM a reliable model for predicting and optimizing adsorption performance in wastewater treatment. This conclusion offers critical insights into the mechanism and initial extraction process during the final extraction phase, aligning with other studies that identified the second-order kinetic model as appropriate for characterizing removal processes^91^. Furthermore, the study supports the use of PSOM in modeling and forecasting the behaviour of removal systems under different operating conditions by highlighting its application in comprehending and improving adsorption processes^92^.

#### Elovich model (EM)

Another kinetic model investigated in the MB dye removal on the NDULB adsorbent is the EM, and the correlation curve between ln (*t*) and *q*_*t*_ is displayed in Fig. 13c. Elovich’s constants *α* and *β* were computed using the slope and intercept of Fig. 13c, respectively. The results are provided in Table 6. After contrasting the *R*^*2*^ values, it can be concluded that, for a specific NDULB dosage, the EM’s *R*^*2*^ values are higher than those of the PFOM and lower than those of the PSOM (Tables 5 and 6). The data in Tables 5 and 6 show that, in some cases, chemical adsorption can regulate the pace at which MB dye adsorbs on NDULB adsorbent.

#### Intraparticle diffusion model (IPDM)

Table 6; Fig. 13d, e compare film diffusion and intraparticle kinetics for MB dye removal using NDULB at different doses. These models are essential to characterizing the mass transfer mechanisms involved in the removal process^40,93–100^. The rate at which the methylene blue passes through the boundary layer and reaches the surface of the adsorbent is indicated by the intraparticle diffusion rate constant (*K*_dif_) (Fig. 13d). *K*_*dif*_ increases with higher initial dye concentrations, for example, rising from 1.633 at 100 ppm to 2.585 at 200 ppm when using 0.5 g L⁻¹ of NDULB. The intercept (*C*) represents the boundary layer thickness, with higher values indicating a stronger influence of surface diffusion. For instance, at 0.5 g L⁻¹ NDULB, *C* increases from 176.12 to 300.17 as the dye concentration rises from 100 to 200 ppm. The high R² values (close to 1.000) confirm that the intraparticle diffusion model effectively describes the adsorption process under most conditions.

#### Film diffusion model (FDM)

The mass transfer rate of methylene blue dye over the liquid film encircling the NDULB particles is reflected by the film diffusion coefficient (*K*_FD_) (Fig. 13e). As the methylene blue dye concentration rises from 100 to 200 ppm at the NDULB dosage of 0.5 g L^–1^, *K*_FD_ varies from 0.047 to 0.012, suggesting that the film diffusion rate may reduce at higher methylene blue dye concentrations. The adsorption kinetics of MB dye onto NDULB are significantly influenced by film diffusion, as suggested by the excellent fit and high *R*^*2*^ values under all circumstances, especially for the film diffusion model. The film diffusion model exhibits an *R*^*2*^ of 0.991 for a methylene blue dye concentration of 200 ppm at an NDULB dosage of 1.5 g L^–1^, suggesting that film diffusion is an important stage in adsorption regulation. Under the same circumstances, the intraparticle diffusion *R*^*2*^ is lower (0.953), suggesting that although intraparticle diffusion aids in the process, film diffusion may be the rate-limiting phase.

**Table 5:** PFOM and PSOM kinetic model results of adsorption of MB dyes by NDULB adsorbent [Initial concentration = (100–200 ppm), NDULB doses = (0.50–1.50 g L^−1^), and Temperature = (25 °C)].

Parameter			PFOM			PSOM			
NDULB **(g L**^**−1**^**)**	**MB dye (ppm)**	***q***_***e***_ **(exp.)**	***q***_***e***_ **(calc.)**	***k***_***1***_ **× 10**^**3**^	**R** ^**2**^	***q***_***e***_ **(calc.)**	***k***_***2***_ **× 10**^**3**^	***h***	**R** ^**2**^
0.50	100	194.34	27.334	46.981	0.978	196.078	4.817	185.19	1.000
	125	219.34	37.094	11.745	0.976	222.222	1.184	58.48	0.999
	150	268.34	43.421	17.042	0.947	270.270	1.256	91.74	0.999
	175	314.47	30.123	9.903	0.950	312.500	1.463	142.86	1.000
	200	335.82	28.774	11.745	0.951	333.333	1.698	188.68	1.000
0.75	100	129.99	14.204	64.945	0.961	129.870	14.117	238.095	1.000
	125	155.40	34.340	15.660	0.974	156.250	1.506	36.765	0.999
	150	186.06	32.240	16.582	0.997	188.679	1.672	59.524	1.000
	175	215.00	28.596	16.351	0.982	217.391	1.873	88.496	1.000
	200	243.12	31.089	14.970	0.960	243.902	1.910	113.636	1.000
1.00	100	97.56	4.801	62.642	0.979	98.039	49.543	476.190	1.000
	125	119.09	22.631	24.872	0.989	120.482	3.131	45.455	1.000
	150	143.77	21.538	21.418	0.990	144.928	3.195	67.114	1.000
	175	167.19	23.046	17.273	0.987	169.492	2.469	70.922	1.000
	200	190.47	27.759	17.273	0.978	192.308	2.292	84.746	1.000
1.25	100	78.11	1.465	47.902	0.928	78.125	102.400	625.000	1.000
	125	97.05	12.070	31.551	0.986	98.039	7.539	72.464	1.000
	150	115.99	17.406	33.394	0.995	117.647	5.432	75.188	1.000
	175	135.44	14.757	22.648	0.977	136.986	4.801	90.090	1.000
	200	154.38	22.506	21.648	0.993	156.250	2.885	70.423	1.000
1.50	100	65.31	0.787	33.163	0.976	65.359	130.050	555.556	1.000
	125	81.35	7.206	29.248	0.932	81.967	12.403	83.333	1.000
	150	96.66	12.471	49.745	0.974	97.087	11.532	108.696	1.000
	175	113.25	8.291	33.624	0.972	113.636	13.586	175.439	1.000
	200	128.82	15.467	21.648	0.991	129.870	4.458	75.188	1.000

**Table 6:** EM, IPDM, and FDM kinetic model results of adsorption of MB dyes by NDULB adsorbent [Initial concentration (100–200 ppm), NDULB doses (0.50–1.50 g L^−1^), and Temperature (25 °C)].

NDULB (g L^−1^)	Parameter		EM				IPDM				FDM	
	MB dye (ppm)	*q*_*e*_ (exp.)	*α*	*β*	R^2^		*K*_*dif*_	*C*	R^2^		*K*_*FD*_	R^2^
0.50	100	194.34	2 × 10^16^	0.21	0.962		1.633	176.12	0.963		0.047	0.978
	125	219.34	2 × 10^5^	0.07	0.984		3.500	173.19	0.990		0.012	0.976
	150	268.34	5 × 10^6^	0.07	0.980		3.132	226.63	0.991		0.015	0.975
	175	314.47	1 × 10^10^	0.08	0.944		2.730	276.68	0.970		0.011	0.968
	200	335.82	6 × 10^15^	0.12	0.955		2.585	300.17	0.983		0.012	0.951
0.75	100	129.99	2 × 10^21^	0.41	0.973		0.808	121.26	0.900		0.065	0.961
	125	155.40	4 × 10^4^	0.09	0.992		2.855	118.36	0.991		0.016	0.974
	150	186.06	1 × 10^8^	0.12	0.993		2.735	151.34	0.987		0.019	0.980
	175	215.00	3 × 10^7^	0.09	0.984		2.239	185.47	0.987		0.016	0.982
	200	243.12	5 × 10^9^	0.10	0.973		2.565	208.44	0.995		0.016	0.975
1.00	100	97.56	1 × 10^40^	1.00	0.944		0.340	94.05	0.806		0.063	0.979
	125	119.09	1 × 10^7^	0.16	0.991		2.104	94.86	0.974		0.025	0.989
	150	143.77	7 × 10^8^	0.16	0.991		1.629	123.25	0.972		0.022	0.987
	175	167.19	7 × 10^10^	0.17	0.986		1.887	142.93	0.993		0.017	0.987
	200	190.47	1 × 10^8^	0.11	0.983		2.456	159.55	0.937		0.017	0.978
1.25	100	78.11	5 × 10^90^	2.75	0.961		0.081	77.23	0.921		0.059	0.976
	125	97.05	1 × 10^10^	0.28	0.973		0.831	87.04	0.944		0.031	0.986
	150	115.99	1 × 10^9^	0.21	0.982		1.186	102.84	0.920		0.033	0.995
	175	135.44	1 × 10^13^	0.25	0.989		1.378	119.33	0.971		0.025	0.993
	200	154.38	1 × 10^8^	0.14	0.988		2.112	130.66	0.992		0.022	0.993
1.50	100	65.31	5 × 10^113^	4.11	0.975		0.039	64.81	0.960		0.033	0.976
	125	81.35	1 × 10^12^	0.39	0.961		0.591	74.16	0.921		0.029	0.941
	150	96.66	7 × 10^11^	0.32	0.948		1.403	84.82	0.906		0.042	0.986
	175	113.25	1 × 10^16^	0.36	0.965		0.731	104.42	0.879		0.035	0.984
	200	128.82	6 × 10^10^	0.22	0.970		1.389	111.83	0.953		0.022	0.991

### Comparison with results reported in the literature

Several earlier investigations on removing methylene blue ions from aqueous solutions are included in Table 7. The NDULB adsorbent has the greatest *Q*_m_ among the publications listed in Table 7, which shows the maximum adsorption capacity (*Q*_m_) for methylene blue dye at room temperature. For the removal of MB dye ions at a concentration of 1.25 g L^–1^ of NDULB, this value was 966.31 mg g^–1^. This study demonstrates that NDULB, which is generated from GAUL, was a very effective adsorbent for eliminating methylene blue dye from wastewater.

**Table 7:** Comparing the highest MB dye adsorption capabilities of different adsorbents.

Adsorbent name	Maximum capacity (mg g^−1^)	Ref.
Co-pyrolysis of lignin and sewage sludge (SS)	154.06	^12^
Activated carbon of *Coriandrum sativum*	94.97	^13^
Sawdust biochar-O_3_-TETA (SDBT)	568.16	^34^
Gigantochloa Bamboo-Derived Biochar	86.60	^46^
Metal − organic framework (MOF) ZJU-48	582.44	^65^
V_2_CT_x_ MXene	111.11	^79^
Surfactant-modified Activated Carbon (SLS-C)	232.50	^86^
Yellow River sediment	520.95	^88^
Cashew nut shell-derived activated carbon	456.00	^92^
NDULB	**966.31**	**This work**

### ANN modeling

**Fig. 14:**
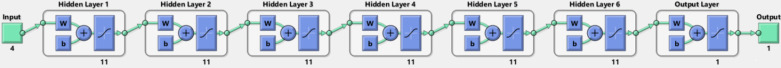
ANN architecture for MB dye adsorption.

**Fig. 15:**
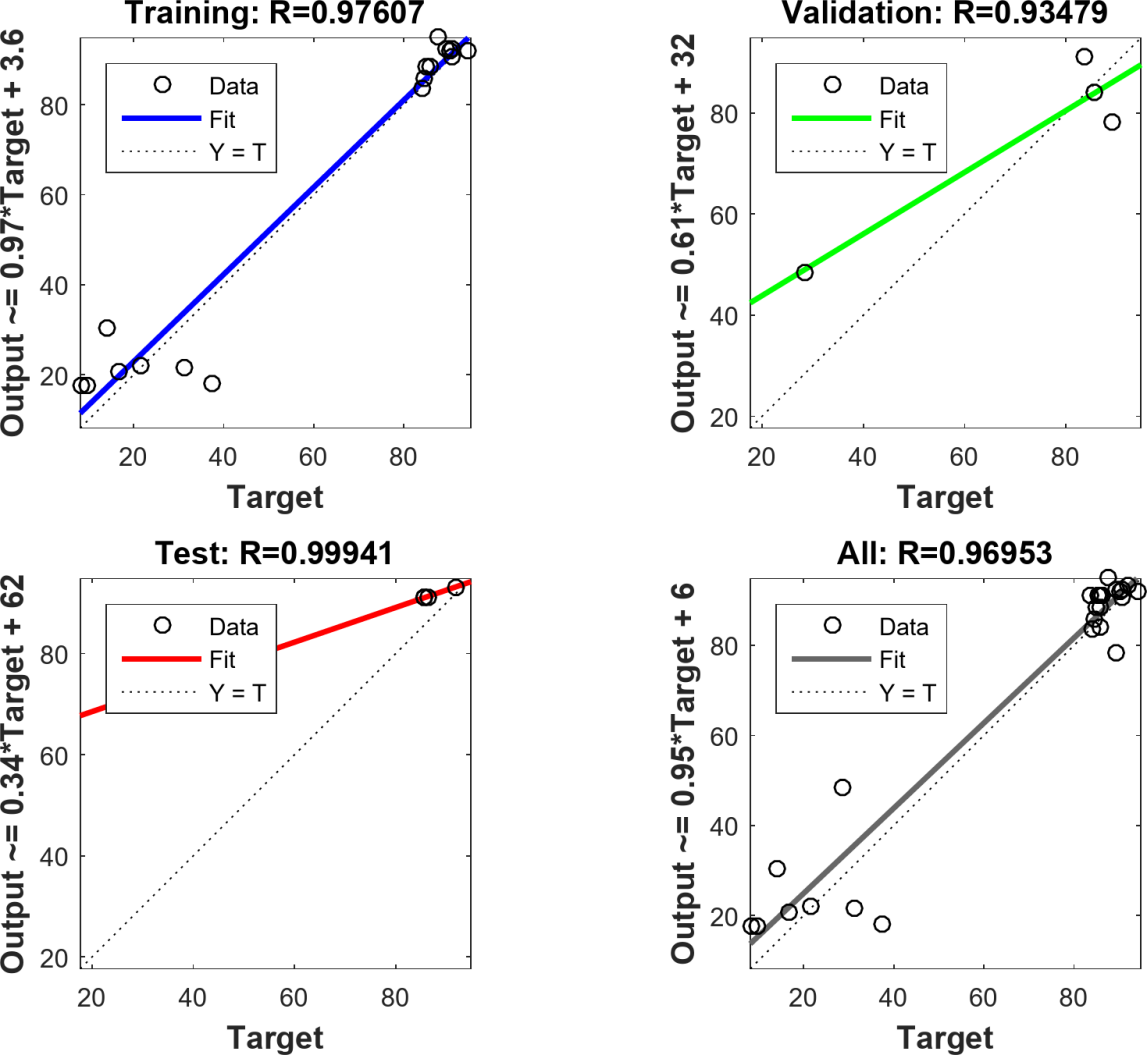
Datasets used for the LM algorithm’s training, validation, testing, and overall applications.

**Fig. 16:**
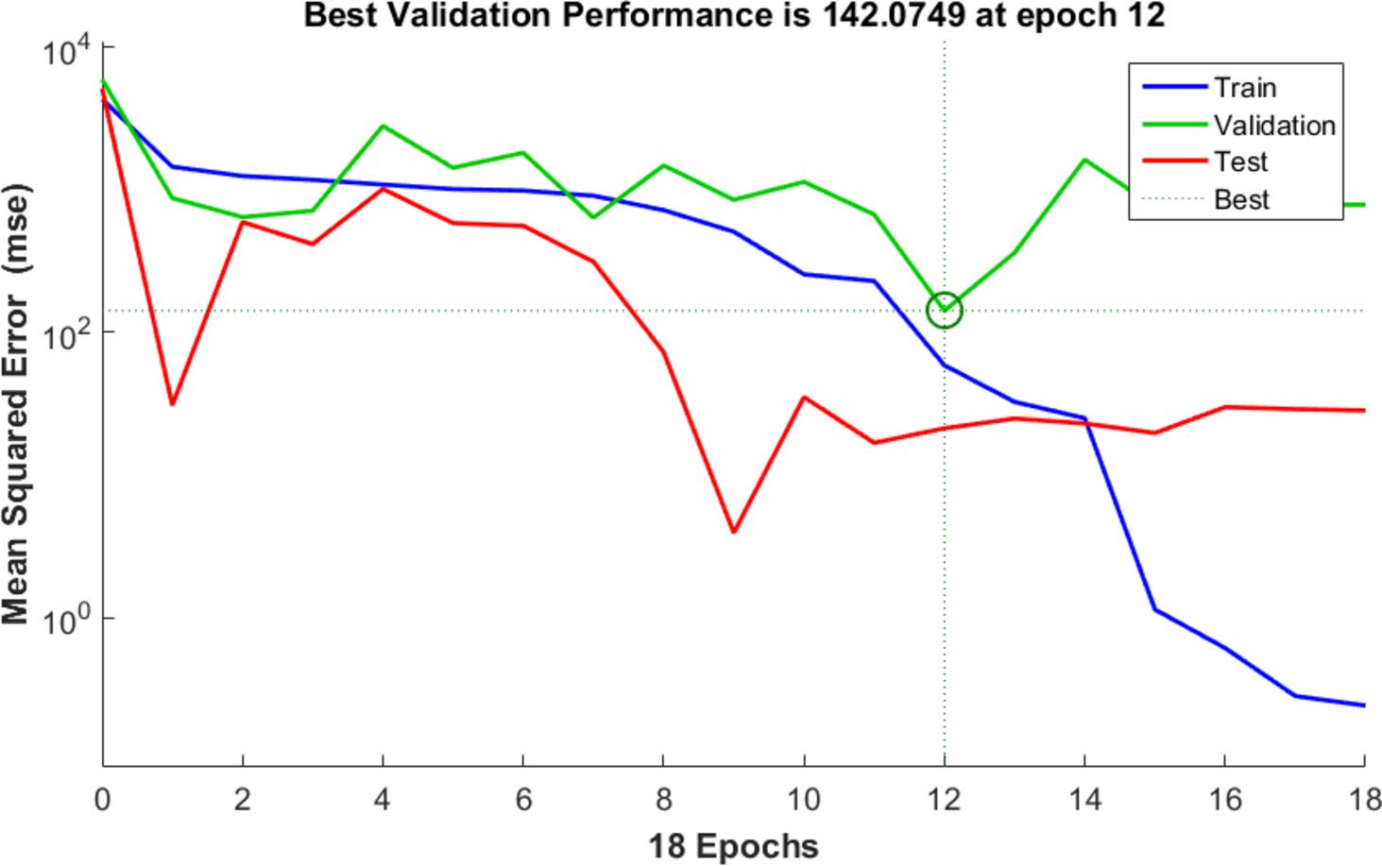
LM algorithm performance.

To assess the optimal ANN model, 70% of the sample data is allocated for training, with the remaining 30% used for validation and testing. The backpropagation algorithm was the training algorithm used to get the MB dye adsorption best-fit ANN model. The optimal ANN model for MB dye adsorption by NDULB was (4-ILs, 11-1st HLs, 11-2nd HLs, 11-3rd HLs, 11-4th HLs, 11-5th HLs, 11-6th HLs, and 1 OL). The architecture of the optimal ANN is shown in Fig. 14. The primary characteristics of the optimal ANN model were the highest *R*^2^ and lowest MSE error values. The regression plots are shown in Fig. 15. The *R*^2^ value for training was 0.97607. The *R*^2^ values for validation and testing were 0.93479 and 0.99941, respectively, and 0.96953 was the overall *R*^2^value. The MSE value was 0.243. The adsorbent dosage of the (NDULB) (mg), the pH of the MB dye, the starting concentration of the MB dye, and the time (min) were the 4 inputs. The only output variable was MB dye removal. Log-Sigmoid (log-sig) for the 1st, 2nd, and 3rd hidden layers, Tan-Sigmoid (tan-sig) for the 4th, 5th, and 6th hidden layers, and purely (linear) for the output layer were the activation functions of the MB dye adsorption best-fit ANN model^101^. Figure 16represented the results of the MSE error vs. the epoch number, which was a sign of the best validation performance of the MB dye adsorption by NDULB optimal ANN model with 12 epochs^102^.

## Conclusion

This study presents the synthesis of a novel adsorbent, green algae (Ulva lactuca)-derived biochar-ammonia (NDULB), and investigates its potential for methylene blue (MB) dye removal from aqueous solutions. The successful characterization of NDULB, coupled with a comprehensive analysis of key parameters such as adsorbent dose, pH, contact time, and initial MB dye concentration, highlights the effectiveness of NDULB as an adsorbent. Green algae (*Ulva lactuca*) biochar-ammonia (NDULB) was obtained from Green algae (*Ulva lactuca*) by being treated with H_2_SO_4_ and Ammonium hydroxide (NH_4_OH) in aqueous solutions. Investigating MB dye’s adsorption effectiveness from aqueous solutions onto NDULB provides important new information about the complex dynamics of methylene blue removal onto NDULB. The Freundlich isotherm model provided the best fit for the adsorption data, suggesting multilayer adsorption on a heterogeneous surface. Kinetic studies indicated that the pseudo-second-order model most accurately describes the adsorption process, supporting a chemisorption mechanism. With a maximum adsorption capacity of 966.31 mg g^–1^ at an NDULB dose of 1.25 g L^–1^ and superior performance across varying conditions, NDULB is shown to be a highly efficient and sustainable adsorbent for MB dye removal. These in-depth understandings are crucial for the development and refinement of large-scale adsorption systems that efficiently remove methylene blue (MB) from effluent. These findings contribute valuable insights into the development of effective and scalable water treatment systems and underscore the importance of optimizing operational parameters in adsorption-based wastewater treatment applications. Future research should further explore the long-term stability and reusability of NDULB in large-scale applications.

## Data Availability

The datasets used in this study are available for review upon request from the corresponding author.
